# Changing epidemiology of inflammatory bowel disease in children and adolescents

**DOI:** 10.1007/s00384-024-04640-9

**Published:** 2024-05-18

**Authors:** Dan Long, Chenchen Wang, Yingtao Huang, Chenhan Mao, Yin Xu, Ying Zhu

**Affiliations:** 1 The First Hospital of Hunan University of Chinese Medicine, Changsha, Hunan China; 2 Hunan University of Chinese Medicine, Changsha, Hunan China; 3 The First Clinical Medical College, Liaoning University of Traditional Chinese Medicine, Shenyang, Liaoning, China; 4 Affiliated Hospital of Integrated Traditional Chinese and Western Medicine, Nanjing University of Chinese Medicine, Nanjing, Jiangsu China

**Keywords:** Inflammatory bowel disease, Global burden of disease, Children and adolescents, Epidemiology

## Abstract

**Background:**

The incidence of inflammatory bowel disease (IBD) is rising worldwide, but epidemiological data on children and adolescents are lacking. Understanding the global burden of IBD among children and adolescents is essential for global standardization of methodology and treatment options.

**Methods:**

This is a cross-sectional study based on aggregated data. We estimated the prevalence and incidence of IBD in children and adolescents between 1990 and 2019 according to the Global Burden of Disease Study 2019 (GBD 2019). Age-standardized rates (ASRs) and estimated annual percentage changes (EAPCs) were used to compare the burden and trends between different regions and countries.

**Results:**

In 2019, there were 25,659 new cases and 88,829 prevalent cases of IBD among children and adolescents globally, representing an increase of 22.8% and 18.5%, respectively, compared to 1990. Over the past 30 years, the incidence and prevalence of IBD among children and adolescents have been highest in high SDI regions, with the most significant increases in East Asia and high-income Asia Pacific. At the age level, incidence and prevalence were significantly higher in the 15–19-year-old age group, while the < 5-year-old group showed the most significant increase in incidence and prevalence.

**Conclusion:**

The incidence of IBD in children and adolescents is significantly on the rise in some countries and regions, and IBD will remain an important public health issue with extensive healthcare and economic costs in the future. The reported IBD burden in children and adolescents at the global, regional, and national levels will assist in the development of more precise health policies.

**Supplementary Information:**

The online version contains supplementary material available at 10.1007/s00384-024-04640-9.

## Introduction

Inflammatory bowel disease (IBD), including Crohn’s disease and ulcerative colitis, is a chronic relapsing immune-mediated disease [1]. There has been an increase in the incidence of IBD, and the age of onset has become younger [2, 3]. Approximately 10–20% of newly diagnosed IBD patients are under 18 years old [4]. Children and adolescents with IBD are likely to experience more severe intestinal involvement and faster disease progression when diagnosed compared to adults [5]. Furthermore, being diagnosed with IBD in childhood presents unique challenges, such as the psychological impact on the child and parents, school absences, and growth retardation [6]. IBD in children and adolescents not only has a highly negative impact on health, but also places a huge economic burden on modern healthcare systems [7].

Previous studies reported that IBD has become a global disease with increasing incidence in newly industrialized countries with more westernized societies [8, 9]. There is significant variation in burdens and trends of IBD in different countries and regions. Recent systematic reviews of IBD in adults described a rapid increase in the incidence of IBD in several countries and regions [8, 10]. Unfortunately, the epidemiological data on IBD among children and adolescents are lacking.

Thus, it is imperative to monitor specific IBD burdens and trends among children and adolescents throughout the world to globally standardize methodology and treatment options. The Global Burden of Disease Study 2019 (GBD 2019) includes health statistics from more than 200 countries, and researchers iterate on the latest data and research techniques, with estimates updated throughout the time series [11]. It is currently the most comprehensive and systematic database on the burden of disease globally and has been widely adopted for disease burden studies [12]. Based on the most recent GBD 2019 estimates, we systematically described the long-term trends in the burden of IBD among children and adolescents at global, regional, and national levels between 1990 and 2019, including prevalence and incidence.

## Materials and methods

### Data sources

This is a cross-sectional study based on aggregated data. This study adhered to the reporting recommendation of the Equator Network (https://www.equator-network.org/). Children and adolescents aged 0 to 19 years were included in our analysis. All data for this study were obtained from the GBD study 2019 database [11]. The search was done through the official website of IHME (https://ghdx.healthdata.org/) utilizing the GBD result tool. The GBD study aims to provide a comprehensive evaluation of the burden caused by major diseases, injuries, and associated risk factors on a global and regional scale. The GBD 2019 assessed the prevalence, incidence, mortality, and disability of 369 diseases by location, sex, age, and year. Detailed descriptions of general methods of GBD 2019 have been described in previous publications [13, 14]. In brief, the GBD Study collected data from censuses, household surveys, civil registration and vital statistics, disease registries, health service use, satellite imaging, disease notifications, and other sources. In identifying these data sources, GBD 2019 conducted a comprehensive assessment of published studies, browsed websites of government and international organizations, analyzed primary data sources like the Demographic and Health Surveys, and incorporated datasets from GBD collaborators. For the GBD 2019 assessment, IBD was defined according to the International Statistical Classification of Diseases and Related Health Problems, Tenth Revision (ICD10) codes (K50–K51.319, K51.5–K52, K52.8–K52.9). In this study, data related to IBD from 1990 to 2019 were stratified by SDI, gender, and age group. Based on the WHO definition and previous studies [15, 16], we divided children and adolescents into four age groups: younger children (< 5 years), older children (5–9 years), younger adolescents (10–14 years), and older adolescents (15–19 years).

### Statistical analysis

As a systematic public health project, GBD 2019 carefully built models and statistical estimates for health loss due to illness, injury, and risk factors based on empirical data. DisMod-MR 2.1, a Bayesian meta-regression modelling tool, was used to model and derive estimates of the burden by age, sex, location, and year. In addition, DisMod-MR 2.1 was designed to overcome the limitations of descriptive epidemiological data, such as missing data, inconsistency, as well as any large variation in methodology between data sources. Firstly, we focused on analyzing prevalent cases, age-standardized prevalence rates (ASPRs), incident cases, and age-standardized incidence rates (ASIRs) of IBD in children and adolescents at global, regional, and national levels. Secondly, we also analyzed the relationship between SDI and IBD burden in children and adolescents. The SDI ranges from 0 to 1, with 0 indicating that the region has the lowest theoretical level of development related to health outcomes, and an SDI value of 1 indicating that the region has the highest theoretical level of development related to health outcomes. Countries are categorized into five levels of low, low-middle, middle, high-middle, and high SDI countries in ascending order according to their SDI values [17].

To reflect trends in IBD burden, we used linear regression analysis to calculate the estimated annual percentage changes (EAPCs) of ASRs [18]. The ASIR is the incidence rate after excluding the effect of age. The ASIR does not reflect the actual incidence rate of IBD, but is only used to compare the incidence rate of IBD in different countries, regions, or periods of history, to allow for comparison of the data [18]. EAPC is a brief and widely used measure of ASR trend over a specific time interval. The natural logarithm of the ASR was assumed to be linear along with time; that is, y = α + βx + ɛ, where y = ln (ASR), x = calendar year, and ε = error term. The EAPC was calculated as 100 × (exp(β) − 1) and its 95% confidence interval (CI) was also obtained from the linear regression model [19]. The ASR was recognized to be in an upward trend if the EAPC estimation and the lower boundary of its 95% CI were both > 0. In contrast, the ASR was in a downward trend if the EAPC estimation and the upper boundary of its 95% CI were both < 0. Otherwise, the ASR was deemed to be stable over time.

R 4.3.1 was used in this study for data analysis and plotting. *P* < 0.05 was considered statistically significant.

## Results

### Global trends

Globally, incident cases of IBD in children and adolescents increased from 20,897 (95% CI: 16,747 to 25,988) in 1990 to 25,659 (20,744 to 31,574) in 2019, an increase of 22.8% (Table 1). Prevalent cases in 2019 were 88,829 (72,096 to 109,441), an increase of 18.5% over 1990 (Table 2). Overall, the incidence and prevalence of IBD among children and adolescents stabilized between 1990 and 2019 (Tables 1 and 2). The incidence and prevalence in 2019 were 0.95 (0.77 to 1.17) and 3.28 (2.66 to 4.05) per 100,000, respectively.

**Table 1:** Incident and prevalent cases, ASIR, ASPR, and EAPC of IBD among children and adolescents globally and in different SDI areas

	Incidence					Prevalence				
	1990		2019		1990–2019	1990		2019		1990–2019
	Cases NO. (95%CI)	ASR/100,000 (95% CI)	Cases NO. (95%CI)	ASR/100,000 (95% CI)	EAPC (95%CI)	Cases NO. (95%CI)	ASR/100,000 (95% CI)	Cases NO. (95%CI)	ASR/100,000 (95% CI)	EAPC (95%CI)
Global	20897.42 (16746.6 to 25987.94)	0.92 (0.74 to 1.15)	25658.55 (20743.87 to 31573.59)	0.95 (0.77 to 1.17)	0.05 (-0.09 to 0.19)	74975.52 (60432.1 to 93339.64)	3.30 (2.66 to 4.10)	88828.9 (72096.12 to 109440.54)	3.28 (2.66 to 4.05)	-0.06 (-0.22 to 0.10)
SDI										
High	10935.98 (9067.78 to 13195.68)	4.25 (3.52 to 5.13)	13913.9 (11676.5 to 16551.99)	5.68 (4.77 to 6.77)	0.85 (0.65 to 1.05)	37811.69 (31799.27 to 45417.37)	14.44 (12.15 to 17.35)	45514.8 (38454.27 to 53719)	18.36 (15.51 to 21.69)	0.69 (0.48 to 0.9)
High-middle	4885.82 (3874.69 to 6097.35)	1.13 (0.9 to 1.42)	4710.52 (3769.11 to 5828.6)	1.34 (1.07 to 1.66)	0.56 (0.50 to 0.62)	18859.9 (14966.19 to 23604.39)	4.32 (3.43 to 5.41)	17785.53 (14270.27 to 22128.41)	5.00 (4.01 to 6.22)	0.49 (0.42 to 0.56)
Middle	2788.36 (2018.74 to 3774.75)	0.35 (0.26 to 0.48)	3385.45 (2469.94 to 4560.83)	0.43 (0.32 to 0.58)	0.69 (0.50 to 0.88)	10461.21 (7614.48 to 14108.97)	1.31 (0.95 to 1.77)	12757.24 (9418.59 to 17047.05)	1.62 (1.19 to 2.16)	0.75 (0.52 to 0.98)
Low-middle	1690.07 (1194.98 to 2354.59)	0.32 (0.22 to 0.44)	2327.06 (1650.69 to 3225.56)	0.32 (0.22 to 0.44)	0.09	5830.01 (4180.33 to 8069.39)	1.10 (0.79 to 1.52)	8238.56 (5873.69 to 11311.05)	1.11 (0.79 to 1.52)	0.12 (0.01 to 0.24)
Low	589.16 (402.62 to 829.94)	0.23 (0.16 to 0.32)	1311.56 (897.81 to 1845.56)	0.23 (0.16 to 0.32)	0.07 (0.04 to 0.11)	1984.55 (1358.81 to 2817.95)	0.79 (0.54 to 1.13)	4499.68 (3074.19 to 6386.48)	0.8 (0.55 to 1.13)	0.11 (0.06 to 0.15)

**Table 2:** Incident and prevalent cases, ASIR, ASPR, and EAPC of IBD among children and adolescents in 21 GBD regions

	Incidence					Prevalence				
	1990		2019		1990–2019	1990		2019		1990–2019
	Cases NO. (95%CI)	ASR/100,000 (95% CI)	Cases NO. (95%CI)	ASR/100,000 (95% CI)	EAPC (95%CI)	Cases NO. (95%CI)	ASR/100,000 (95% CI)	Cases NO. (95%CI)	ASR/100,000 (95% CI)	EAPC (95%CI)
Andean Latin America	49.54 (34.55 to 68.46)	0.27 (0.19 to 0.37)	71.17 (51.3 to 96.69)	0.30 (0.21 to 0.4)	0.12 (0.01 to 0.24)	178.94 (125.03 to 248.7)	0.97 (0.68 to 1.35)	257.7 (183.69 to 349.52)	1.06 (0.76 to 1.44)	0.22 (0.19 to 0.26)
Australasia	184.56 (142.68 to 234.14)	2.64 (2.04 to 3.35)	363.18 (291.41 to 452.72)	4.81 (3.86 to 6)	1.73 (1.31 to 2.16)	636.74 (491.51 to 803.58)	8.91 (6.88 to 11.25)	1127.67 (898.73 to 1398.85)	14.90 (11.87 to 18.48)	1.49 (1.09 to 1.89)
Caribbean	68.33 (50.3 to 91.91)	0.44 (0.32 to 0.59)	73.22 (52.85 to 100.08)	0.45 (0.32 to 0.61)	0.02 (-0.01 to 0.05)	269.04 (198.08 to 357.42)	1.71 (1.26 to 2.28)	257.54 (186.41 to 346.9)	1.56 (1.13 to 2.1)	-0.17 (-0.24 to -0.1)
Central Asia	277.12 (206.34 to 364.69)	0.94 (0.7 to 1.23)	323.12 (241.67 to 425.55)	1.00 (0.75 to 1.32)	0.24 (0.22 to 0.26)	1137.48 (849.24 to 1493.53)	3.87 (2.89 to 5.08)	1271.79 (952.2 to 1679.99)	3.99 (2.99 to 5.27)	0.10 (0.09 to 0.11)
Central Europe	1289.42 (1016.92 to 1620.5)	3.07 (2.42 to 3.86)	999.53 (812.13 to 1224.22)	3.99 (3.24 to 4.89)	0.90 (0.77 to 1.03)	5549.48 (4458.44 to 6930.26)	13.12 (10.54 to 16.39)	4314.59 (3547.72 to 5263.85)	17.09 (14.05 to 20.86)	0.97 (0.81 to 1.12)
Central Latin America	371.38 (270.84 to 497.74)	0.46 (0.33 to 0.61)	408.93 (298.47 to 548.85)	0.44 (0.32 to 0.59)	-0.21 (-0.29 to -0.13)	1317.82 (962.54 to 1761.26)	1.63 (1.19 to 2.17)	1561.16 (1145.6 to 2085.12)	1.65 (1.21 to 2.2)	0.06 (0.02 to 0.10)
Central Sub-Saharan Africa	49.62 (32.06 to 70.77)	0.18 (0.12 to 0.26)	132.81 (87.34 to 191.1)	0.20 (0.13 to 0.29)	0.26 (0.2 to 0.32)	175.56 (114.67 to 252.75)	0.66 (0.44 to 0.95)	440.71 (289.85 to 642.65)	0.67 (0.44 to 0.98)	-0.03 (-0.14 to 0.09)
East Asia	1720.33 (1222.69 to 2355.04)	0.33 (0.24 to 0.46)	2195.43 (1646.02 to 2871.98)	0.67 (0.5 to 0.88)	2.64 (2.27 to 3.02)	6957.11 (4953.33 to 9468.54)	1.31 (0.93 to 1.78)	8510.98 (6427.52 to 11043.32)	2.56 (1.93 to 3.33)	2.59 (2.16 to 3.03)
Eastern Europe	637.17 (477.27 to 832.26)	0.92 (0.69 to 1.2)	462.14 (348.31 to 599.89)	0.99 (0.75 to 1.28)	0.16 (0.1 to 0.23)	2726.01 (2065.71 to 3546.56)	3.92 (2.97 to 5.10)	1871.21 (1419.62 to 2426.15)	4.03 (3.06 to 5.22)	0.11 (0.1 to 0.12)
Eastern Sub-Saharan Africa	157.46 (103.04 to 227.09)	0.16 (0.11 to 0.23)	373.58 (245.48 to 535.43)	0.17 (0.11 to 0.25)	0.31 (0.27 to 0.35)	543.83 (356.15 to 779.76)	0.57 (0.38 to 0.82)	1298.61 (856.83 to 1868.91)	0.61 (0.40 to 0.88)	0.27 (0.25 to 0.29)
High-income Asia Pacific	1024.53 (781.42 to 1324.49)	1.66 (1.27 to 2.15)	1935.62 (1556.24 to 2393.81)	5.15 (4.14 to 6.38)	2.30 (1.58 to 3.03)	3889.16 (2991.01 to 5009.3)	6.14 (4.72 to 7.91)	6310.66 (5169.39 to 7727.24)	16.55 (13.55 to 20.28)	1.98 (1.30 to 2.65)
High-income North America	6134.08 (5057.95 to 7410.9)	7.18 (5.93 to 8.68)	7659.4 (6511.3 to 8972.21)	7.62 (6.47 to 8.93)	0.40 (0.28 to 0.52)	21632.66 (18116.53 to 25990.11)	25.06 (20.99 to 30.10)	26506.68 (22550.7 to 31008.54)	25.99 (22.1 to 30.41)	0.29 (0.18 to 0.41)
North Africa and Middle East	1125.36 (849.78 to 1467.01)	0.67 (0.51 to 0.87)	1716.45 (1278.81 to 2295.37)	0.74 (0.55 to 0.99)	0.11 (-0.02 to 0.24)	4041.79 (3065.3 to 5264.55)	2.44 (1.85 to 3.18)	6409.85 (4835.43 to 8453.11)	2.75 (2.07 to 3.62)	0.31 (0.23 to 0.38)
Oceania	3.79 (2.38 to 5.48)	0.12 (0.08 to 0.17)	7.79 (4.98 to 11.18)	0.13 (0.09 to 0.19)	0.37 (0.32 to 0.42)	14.59 (9.1 to 21.28)	0.47 (0.29 to 0.69)	26.8 (17.11 to 38.79)	0.47 (0.30 to 0.68)	-0.09 (-0.14 to -0.05)
South Asia	1521.15 (1039.84 to 2190.54)	0.30(0.21 to 0.43)	2164.46 (1491.94 to 3074.66)	0.29 (0.2 to 0.41)	0.02 (-0.15 to 0.19)	5173.08 (3595.87 to 7393.63)	1.04 (0.72 to 1.48)	7624.78 (5302.72 to 10872.27)	1.00 (0.69 to 1.42)	0 (-0.15 to 0.14)
Southeast Asia	322.12 (212.69 to 454.3)	0.15 (0.1 to 0.21)	476.07 (328.73 to 653.82)	0.20 (0.14 to 0.27)	1.04 (0.82 to 1.25)	1256.54 (814.35 to 1786.49)	0.57 (0.37 to 0.81)	1803.91 (1239.78 to 2490.77)	0.74 (0.51 to 1.02)	0.9 (0.68 to 1.12)
Southern Latin America	91.37 (66.61 to 122.02)	0.47 (0.34 to 0.62)	103.27 (75.12 to 137.98)	0.48 (0.35 to 0.65)	0.19 (-0.05 to 0.42)	328.26 (241.06 to 441.73)	1.67 (1.23 to 2.25)	375.41 (276.7 to 504.41)	1.73 (1.27 to 2.33)	0.19 (-0.08 to 0.46)
Southern Sub-Saharan Africa	50.92 (33.79 to 71.75)	0.20 (0.13 to 0.28)	63.15 (42.01 to 89.11)	0.20(0.14 to 0.29)	0.12 (0.1 to 0.14)	194.86 (130.39 to 277.43)	0.75 (0.5 to 1.07)	245.87 (166.37 to 350.47)	0.80 (0.54 to 1.13)	0.26 (0.22 to 0.3)
Tropical Latin America	733.37 (562.73 to 938.3)	1.04 (0.8 to 1.33)	643.33 (489.6 to 829.71)	0.89 (0.67 to 1.15)	-0.40 (-0.47 to -0.34)	2642.7 (2042.26 to 3395.29)	3.76 (2.91 to 4.83)	2408.88 (1835.35 to 3133.84)	3.28 (2.49 to 4.26)	-0.26 (-0.35 to -0.17)
Western Europe	4926.08 (4191.9 to 5796.32)	4.37 (3.71 to 5.15)	5057.24 (4235.75 to 6024.68)	5.01 (4.19 to 5.97)	0.33 (0.17 to 0.48)	15732.64 (13467.6 to 18696.77)	13.65 (11.68 to 16.23)	14597.96 (12224.8 to 17569.21)	14.36 (12.02 to 17.29)	0.06 (-0.12 to 0.24)
Western Sub-Saharan Africa	159.71 (104.71 to 228.25)	0.17 (0.11 to 0.24)	428.66 (282.96 to 605.19)	0.18 (0.12 to 0.26)	0.16 (0.1 to 0.22)	577.23 (379.02 to 829.02)	0.63 (0.42 to 0.91)	1606.14 (1074.79 to 2302.93)	0.70 (0.47 to 1.00)	0.37 (0.34 to 0.41)

### Gender and age patterns

**Fig. 1:**
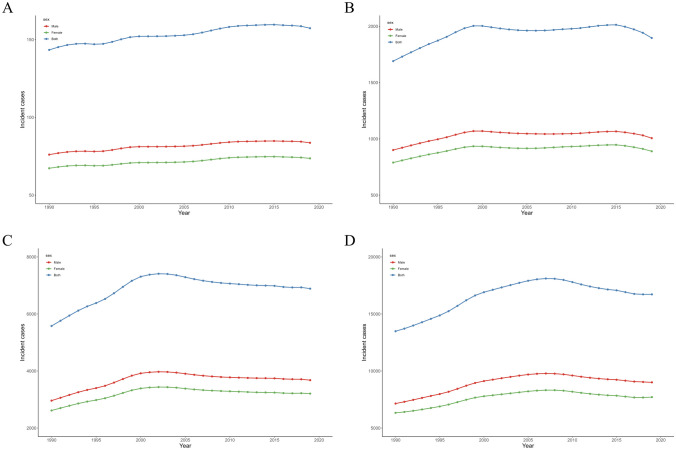
Temporal trend of incident cases of children and adolescents with IBD in different age groups from 1990 to 2019 globally. **A** Temporal trend of incident cases for those aged < 5 years; **B** Temporal trend of incident cases for those aged 5 to 9 years; **C** Temporal trend of incident cases for those aged 10 to 14 years; **D** Temporal trend of incident cases for those aged 15 to 19 years. IBD, inflammatory bowel disease

**Fig. 2:**
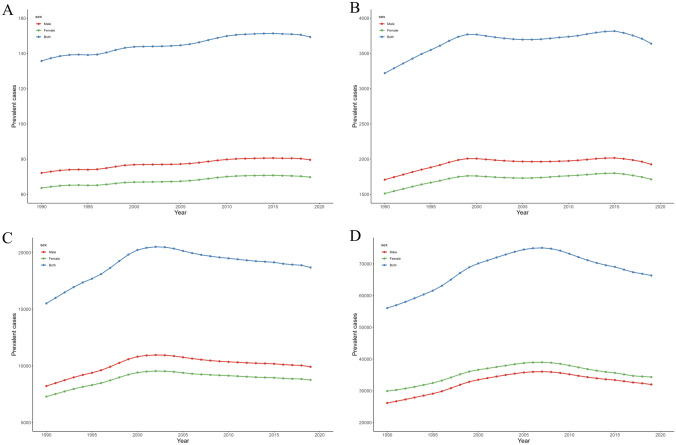
Temporal trend of prevalent cases of children and adolescents with IBD in different age groups from 1990 to 2019 globally. **A** Temporal trend of prevalent cases for those aged < 5 years; **B** Temporal trend of prevalent cases for those aged 5 to 9 years; **C** Temporal trend of prevalent cases for those aged 10 to 14 years; **D** Temporal trend of prevalent cases for those aged 15 to 19 years. IBD, inflammatory bowel disease

Global trends in incidence and prevalence by sex and age group from 1990 to 2019 are shown in Supplementary Table 1 and Figs. 1 to 2. Globally, the incidence and prevalence of IBD in children and adolescents aged 0 to 19 years have remained generally stable in both males and females. The prevalence rates for boys and girls in 2019 were 3.16 (2.56 to 3.92) and 3.41 (2.78 to 4.18) per 100,000, respectively. Whereas the incidence was slightly higher in boys (1 (0.8 to 1.23) per 100,000) than in girls (0.91 (0.74 to 1.11) per 100,000).

The incident cases in older adolescents were significantly higher than any other age group, reaching 16,718 (13,620 to 20,286) in 2019. The incidence rate of older adolescents was also highest (2.7 (2.2 to 3.27) per 100,000). Prevalent cases (66,348 (54,242 to 81,002)) and prevalence rate (10.71 (8.76 to 13.07) per 100,000) were also significantly higher among older adolescents. However, we found that the increasing trend in incidence and prevalence between 1990 and 2019 was only observed in the < 5 years age group, while the remaining three age groups stabilized.

### Association with the sociodemographic index (SDI)

**Fig. 3:**
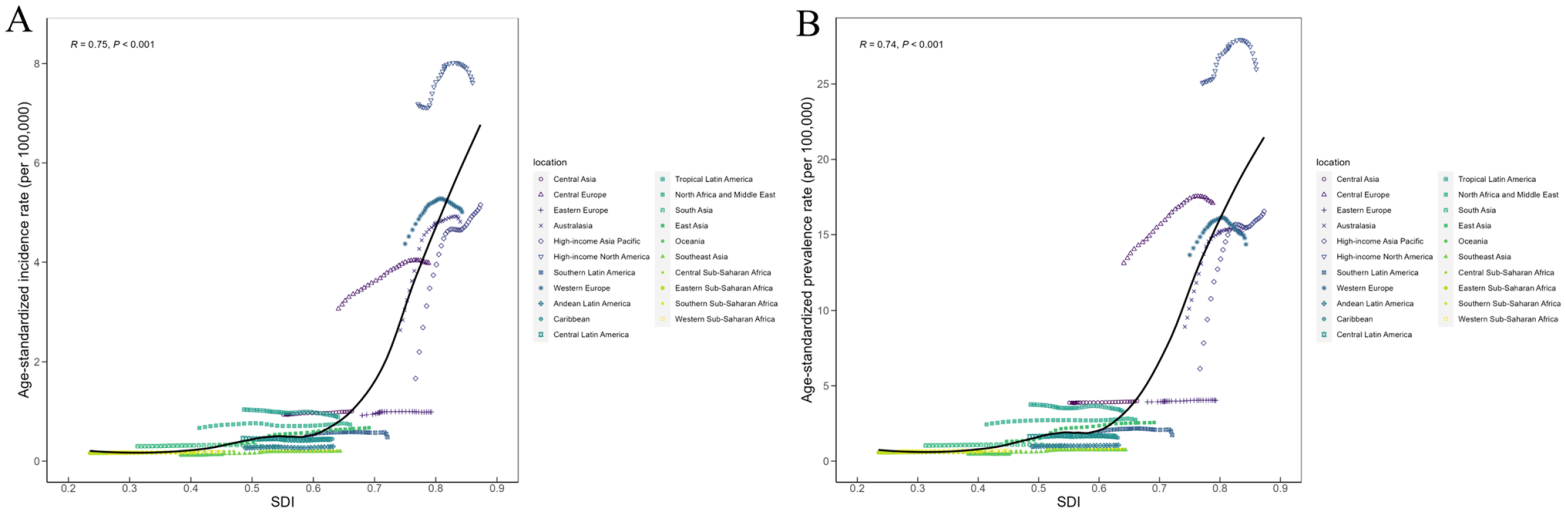
Burden of IBD among children and adolescents in 21 GBD regions by SDI, from 1990 to 2019. **A** ASPR; **B** ASIR. IBD, inflammatory bowel disease; SDI, socio-demographic index; ASPR, age-standardized prevalence rate; ASIR, age-standardized incidence rate

IBD burden among children and adolescents in different SDI regions is presented in Tables 1 and 2. Incidence rates in the low-middle SDI area did not change significantly during the study period, while the remaining four SDI areas all increased. Prevalence increased in all five SDI areas, with the largest rise occurring in the middle SDI areas (EAPC: 0.75 (0.52 to 0.98)). In 2019, incidenct and prevalent cases were about 10 times in high SDI areas compared with low SDI areas. Meanwhile, the highest incidence rate was observed in high SDI areas (5.68 (4.77 to 6.77) per 100,000) in 2019, with low SDI areas (0.23 (0.16 to 0.32) per 100,000) having the lowest estimate. The variation in incidence and prevalence rates across SDI by 21 GBD regions is shown in Fig. 3. As shown in Fig. 3, the incidence and prevalence of IBD in children and adolescents overall are positively correlated with SDI.

### Regional levels

At the regional level, the highest prevalent cases were observed in East Asia (8511 (6428 to 11,043)) and South Asia (7625 (5303 to 10,872)). The largest increases in prevalent cases between 1990 and 2019 were observed in Western Sub-Saharan Africa (increased by 178.2%), Central Sub-Saharan Africa (increased by 151%), and Eastern Sub-Saharan Africa (increased by 138.8%). Notably, East Asia experienced the greatest increase in both incidence and prevalence between 1990 and 2019. The highest incidence rate in 2019 was in high-income North America (7.62 (6.47 to 8.93) per 100,000) and high-income Asia Pacific (5.15 (4.14 to 6.38) per 100,000). The lowest incidence and prevalence rates were observed in Oceania and Eastern Sub-Saharan Africa.

### National levels

**Fig. 4:**
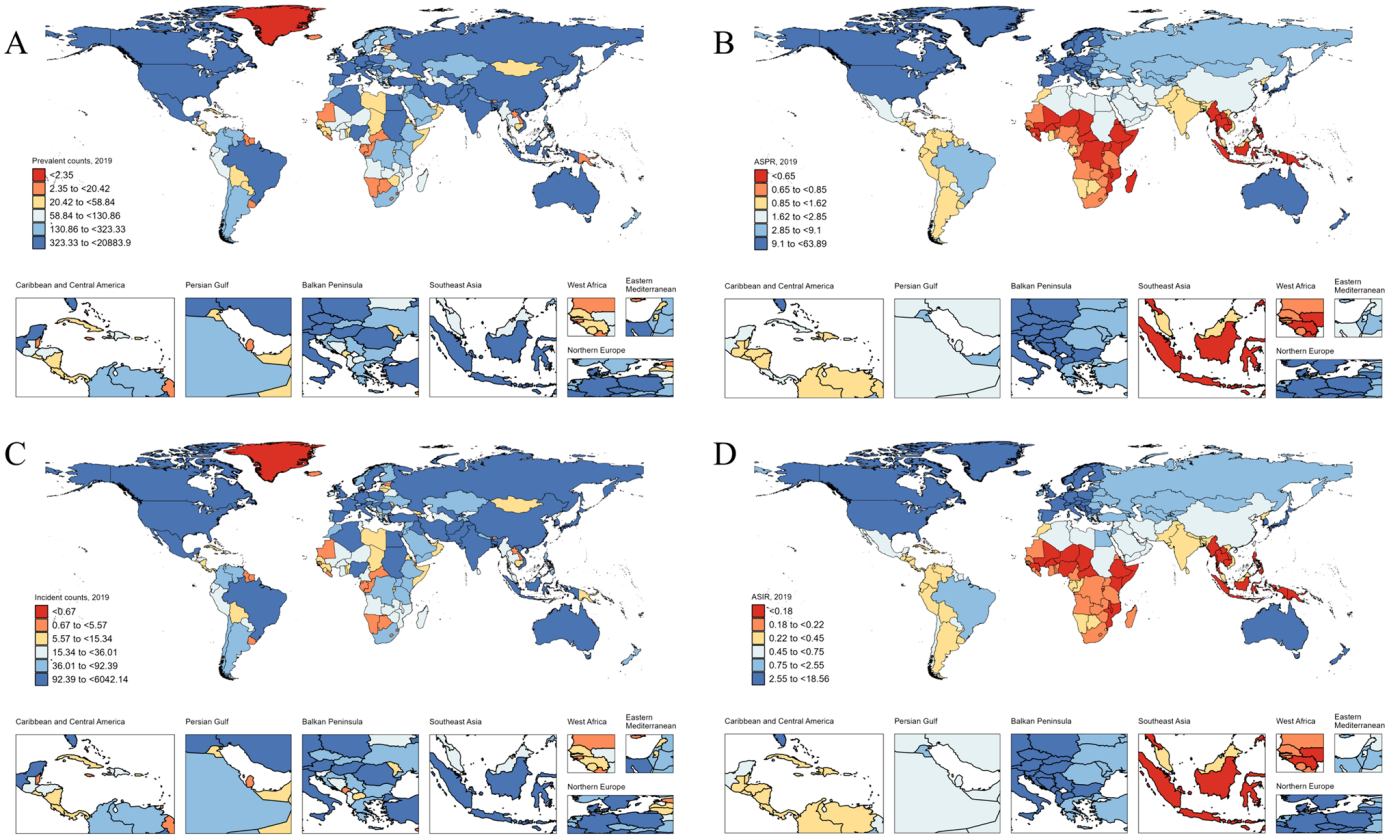
Prevalence and incidence of children and adolescents with IBD in different countries and territories in 2019. **A** Prevalent cases; **B** ASPR; **C** Incident cases; **D** ASIR. IBD, inflammatory bowel disease; ASPR, age-standardized prevalence rate; ASIR, age-standardized incidence rate

IBD burden among children and adolescents in different countries and territories is shown in Supplementary Tables 2 and 3 and Fig. 4. Nationally, the United States of America (20,884), China (8285), and India (5682) showed the highest prevalent cases. We found huge disparities in the burden of IBD in children and adolescents across countries, with differences of more than 140 times in both incidence and prevalence. Notably, the largest increases in incidence and prevalence were recorded in Taiwan (Province of China), Japan, and the Republic of Korea. In 2019, Canada, Denmark, and Hungary showed the highest incidence and prevalence rates. The countries with the lowest ASIRs were Papua New Guinea (0.13 (0.08 to 0.19) per 100,000) and Solomon Islands (0.13 (0.08 to 0.19) per 100,000).

## Discussion

IBD in children usually has faster disease progression and more extensive lesions and also leads to specific complications such as growth retardation and delayed puberty [20]. To our knowledge, this was the first study to describe the incidence and prevalence of IBD among children and adolescents aged less than 19 years, from 1990 to 2019, at the global, regional, and national levels. Despite an increase in the number of incident and prevalent cases of IBD among children and adolescents globally between 1990 and 2019, the incidence and prevalence rates have shown a stable trend. High-income North America and high-income Asia Pacific showed the highest incidence and prevalence rates, which may be related to the risk factors of IBD. Although the pathogenesis of IBD is unclear, some studies have identified its risk factors [21–23]. For example, diet affects the development of IBD by altering the microbiota and modulating immune function [24]. Early life is a critical period for microbiome establishment and immune maturation. The gut microbiota has been demonstrated to play a critical role in the pathogenesis of IBD [24]. Notably, the microbiome interacts with the host immune system during the prenatal, perinatal, and postnatal periods, which may play a key role in the development of IBD [25–28]. The early-life events might alter the development of the gut microbiota. Cesarean delivery and breastfeeding are thought to potentially reduce the risk of IBD [22]. Furthermore, infections within the first year of life have been reported to affect the risk of suffering from IBD later in life [22]. Importantly, a growing number of studies have indicated that antibiotics increase the risk of IBD in a dose-dependent manner across all age groups [29, 30].

We found that the incidence and prevalence of IBD in children and adolescents have shown a significant upward trend in several countries and regions. Notably, East Asia experienced the greatest increase in both incidence and prevalence between 1990 and 2019. In addition, the incidence and prevalence in high-income Asia Pacific and Australasia have also increased significantly. A prospective population-based study in 13 countries or regions in Asia–Pacific suggests that the prevalence of IBD is higher in densely populated areas of Asia and that the prevalence may be associated with increased urbanization [31]. The importance of environmental risk factors in this disease is clear, and hygiene is a key factor [32]. In addition, primary immunodeficiencies may be a risk factor for early-onset IBD in East Asia [33].

Nationally, the United States of America, China, and India showed the highest prevalent cases. We found huge disparities in the burden of IBD in children and adolescents across countries. It is necessary to reduce health inequality by strengthening international cooperation. Canada had the highest ASIR, which is consistent with the findings of a previous study [2]. To better plan the health care system and adequately address the heavy burden of IBD in children and adolescents, continuous monitoring of IBD epidemiology and health service utilization in Canada is necessary. In addition, modifying exposure to environmental risk factors (such as western diets and lifestyles) associated with the westernization of society may provide a pathway to reducing the risk of IBD in Canada [34]. Notably, Taiwan (Province of China) experienced the largest increase in incidence and prevalence, followed by Japan and Republic of Korea. More research is needed to understand the reasons for the increased incidence in these countries [35].

IBD is disabling [36]. Effective inter-country interventions and collaboration should be promoted to improve healthcare for children with IBD in countries with low SDI. The incidence and prevalence of rates were significantly higher in the 15–19 years age group and increased most significantly in the < 5 years age group. Very early-onset inflammatory bowel disease (VEO-IBD) is defined as IBD with onset before the age of 6 years [37]. Notably, patients with VEO-IBD experience more severe disease, are less responsive to conventional treatment, and have a poorer prognosis than older patients [38]. It was found that pediatric-onset IBD may increase the risk of cancer [20, 39]. Therefore, younger children suffering from IBD deserve more attention.

Standardized and precise clinical strategies for the treatment of IBD among children and adolescents should not be overlooked. Nutritional interventions have long been recognized as a key component in the treatment of pediatric IBD [40]. Second, therapeutic drug monitoring is routinely recommended to optimize or monitor IBD therapy. During the use of azathioprine or 6-mercaptopurine or methotrexate, regular monitoring of blood counts and associated adverse effects is necessary. Furthermore, medical therapy is recommended for children after bowel resection for Crohn’s disease who have risk factors for recurrence. Children must be routinely tested for clostridium difficile if they experience a recurrence or exacerbation of diarrhea during follow-up [41]. It is also crucial to avoid known risk factors to reduce the risk of IBD. Health education for children and parents, such as the promotion of proper diet and breastfeeding, should be strengthened.

There are some limitations of this study. First, we assessed IBD burden in children and adolescents based on age, sex, and SDI, and failed to assess other risk factors for IBD due to the lack of risk factor statistics for it in the database. In addition, as the data were aggregated across multiple sites globally, there may be limitations of underdiagnosis and underreporting, which may lead to an underestimation of our results and require caution when interpreting the results. Finally, due to the unavailability of detailed data on ulcerative colitis and Crohn’s disease in the GBD database, we were unable to count the epidemiological situation of both separately in this study.

## Conclusion

Globally, the incidence and prevalence of IBD among children and adolescents showed a stable trend between 1990 and 2019. However, some countries and regions have a significant upward trend in the incidence of IBD among children and adolescents, especially in East Asia. IBD will continue to be an important public health problem with extensive healthcare and economic costs in the future. The reported IBD burden in children and adolescents at the global, regional, and national levels will inform policymakers and help them formulate locally adapted health policies.

## Supplementary Information

Below is the link to the electronic supplementary material.

Supplementary file1 (DOCX 74 KB)

## Data Availability

No datasets were generated or analysed during the current study.
