# Supplementary material for: Changing epidemiology of inflammatory bowel disease in children and adolescents
Source: Int J Colorectal Dis. 2024 May 18;39(1):73. doi: 10.1007/s00384-024-04640-9 (PMC11101569; doi:10.1007/s00384-024-04640-9)
Supplement: Supplementary file 1 — Supplementary file1 (DOCX 74 KB) [file 384_2024_4640_MOESM1_ESM.docx]

**Supplementary Table 1** Global incidence and prevalence of IBD among children and adolescents in different age groups in 1990 and 2019 and the EAPC values

|  | 1990 |  | 2019 |  | 1990-2019 |
| --- | --- | --- | --- | --- | --- |
|  | Cases NO.(95%UI) | ASR/100,000 (95% CI) | Cases NO.(95%UI) | ASR/100,000 (95% CI) | EAPC (95%CI) |
| Incidence |  |  |  |  |  |
| Both |  |  |  |  |  |
| <5 years | 143.36 (93.1 to 204.65) | 0.02 (0.01 to 0.03) | 157.3 (103.02 to 225.07) | 0.02 (0.02 to 0.03) | 0.14 (0.07 to 0.21) |
| 5–9 years | 1690.61 (1306.15 to 2157.09) | 0.29 (0.22 to 0.37) | 1896.22 (1450.02 to 2455.82) | 0.29 (0.22 to 0.38) | 0.07  (-0.09 to 0.24) |
| 10–14 years | 5578.13 (4491.67 to 7011.52) | 1.04 (0.84 to 1.31) | 6887.51 (5570.88 to 8606.81) | 1.07 (0.87 to 1.34) | 0.10 (-0.06 to 0.25) |
| 15–19 years | 13485.33 (10855.69 to 16614.69) | 2.6 (2.09 to 3.2) | 16717.52 (13619.95 to 20285.89) | 2.70 (2.20 to 3.27) | 0.03  (-0.11 to 0.16) |
| Male |  |  |  |  |  |
| <5 years | 76.06 (49.45 to 108.03) | 0.02 (0.02 to 0.03) | 83.66 (55.15 to 118.53) | 0.02 (0.02 to 0.03) | 0.14  (0.07 to 0.21) |
| 5–9 years | 900.81 (697.98 to 1149.61) | 0.3 (0.23 to 0.38) | 1006.35 (770.44 to 1302.6) | 0.3 (0.23 to 0.39) | 0.04  (-0.12 to 0.21) |
| 10–14 years | 2961.6 (2379.93 to 3732.87) | 1.08 (0.87 to 1.36) | 3679.14 (2969.39 to 4599.6) | 1.11 (0.9 to 1.39) | 0.09  (-0.06 to 0.24) |
| 15–19 years | 7149.18 (5740.61 to 8797.28) | 2.71 (2.17 to 3.33) | 9006.91 (7301.08 to 10988.69) | 2.83 (2.3 to 3.46) | 0.03  (-0.12 to 0.18) |
| Female |  |  |  |  |  |
| <5 years | 67.3 (43.12 to 96.8) | 0.02 (0.01 to 0.03) | 73.64 (48.09 to 105.65) | 0.02 (0.02 to 0.03) | 0.15  (0.08 to 0.21) |
| 5–9 years | 789.8 (607.94 to 1009.92) | 0.28 (0.21 to 0.35) | 889.87 (682.03 to 1145.45) | 0.28 (0.22 to 0.36) | 0.11  (-0.06 to 0.28) |
| 10–14 years | 2616.53 (2112.38 to 3261.41) | 1 .00(0.81 to 1.25) | 3208.37 (2594.88 to 3993.81) | 1.03 (0.83 to 1.28) | 0.1  (-0.06 to 0.25) |
| 15–19 years | 6336.15 (5103.21 to 7808.33) | 2.48 (2 to 3.06) | 7710.62 (6327.2 to 9280.53) | 2.56 (2.1 to 3.08) | 0.02  (-0.11 to 0.15) |
| Prevalence |  |  |  |  |  |
| Both |  |  |  |  |  |
| <5 years | 135.76 (87.92 to 194.56) | 0.02 (0.01 to 0.03) | 149.36 (97.48 to 214.13) | 0.02 (0.01 to 0.03) | 0.16 (0.09 to 0.22) |
| 5–9 years | 3220.71 (2471.04 to 4212.29) | 0.55 (0.42 to 0.72) | 3639.31 (2777.4 to 4796.2) | 0.56 (0.42 to 0.73) | 0.09  (-0.06 to 0.24) |
| 10–14 years | 15523.07 (12408.44 to 19451.16) | 2.89 (2.31 to 3.62) | 18692.65 (14979.24 to 23428.37) | 2.91 (2.33 to 3.65) | 0.03  (-0.13 to 0.19) |
| 15–19 years | 56095.97 (45464.7 to 69481.63) | 10.8 (8.75 to 13.37) | 66347.58 (54242 to 81001.84) | 10.71 (8.76 to 13.07) | -0.1  (-0.26 to 0.07) |
| Male |  |  |  |  |  |
| <5 years | 72.14 (47.12 to 103.07) | 0.02 (0.01 to 0.03) | 79.6 (52.11 to 113.07) | 0.02 (0.02 to 0.03) | 0.15  (0.09 to 0.22) |
| 5–9 years | 1708.6 (1315.37 to 2233.35) | 0.57 (0.44 to 0.74) | 1925.97 (1468.43 to 2538.56) | 0.57 (0.43 to 0.75) | 0.06  (-0.09 to 0.21) |
| 10–14 years | 8226.97 (6564.86 to 10326.1) | 2.99 (2.39 to 3.76) | 9924.06 (7940.82 to 12490.87) | 3 (2.4 to 3.77) | 0.02  (-0.14 to 0.18) |
| 15–19 years | 26167 (21104.49 to 32528.09) | 9.91 (7.99 to 12.32) | 32001.98 (26073.01 to 39300.15) | 10.07 (8.2 to 12.37) | -0.03  (-0.2 to 0.15) |
| Female |  |  |  |  |  |
| <5 years | 63.61 (40.75 to 91.71) | 0.02 (0.01 to 0.03) | 69.75 (45.2 to 101.03) | 0.02 (0.01 to 0.03) | 0.16  (0.1 to 0.22) |
| 5–9 years | 1512.12 (1150.2 to 1982.52) | 0.53 (0.4 to 0.7) | 1713.35 (1308.26 to 2258.57) | 0.54 (0.41 to 0.71) | 0.12  (-0.03 to 0.27) |
| 10–14 years | 7296.11 (5843.01 to 9145.45) | 2.79 (2.23 to 3.49) | 8768.59 (7038.42 to 10943.44) | 2.82 (2.26 to 3.52) | 0.05  (-0.11 to 0.21) |
| 15–19 years | 29928.97 (24314.86 to 37067.79) | 11.71 (9.52 to 14.51) | 34345.6 (28267.47 to 41727.03) | 11.38 (9.37 to 13.83) | -0.15  (-0.31 to 0) |

**Supplementary Table 2** Incidence of IBD among children and adolescents in 1990 and 2019 and the EAPC in different countries and territories

| location | 1990 |  | 2019 |  | 1990-2019 |
| --- | --- | --- | --- | --- | --- |
|  | Incidence cases NO.(95%UI) | ASR/100,000 (95% CI) | Incidence cases NO.(95%UI) | ASR/100,000 (95% CI) | EAPC (95%CI) |
| Canada | 1532.41 (1441.65 to 1621.79) | 18.98 (17.54 to 20.52) | 1616.49 (1505.75 to 1719.01) | 18.56 (16.91 to 20.21) | 0.33 (0.15 to 0.52) |
| Denmark | 153.88 (135.25 to 172.98) | 10.31 (8.87 to 11.91) | 161.85 (140.65 to 184.54) | 11.14 (9.42 to 13.06) | 0.46 (0.31 to 0.61) |
| Hungary | 167.56 (143.04 to 193.52) | 4.98 (4.16 to 5.86) | 159.77 (138.63 to 183.78) | 7.67 (6.5 to 9.01) | 1.91 (1.5 to 2.32) |
| Austria | 159.46 (135.5 to 186.1) | 7.47 (6.19 to 8.91) | 141.17 (120.68 to 165.77) | 7.36 (6.08 to 8.9) | 0.11 (-0.24 to 0.45) |
| Norway | 52.05 (40.46 to 67.61) | 4.03 (3.07 to 5.27) | 92.24 (73.44 to 115.84) | 6.66 (5.15 to 8.47) | 0.81 (0.45 to 1.17) |
| Finland | 104 (94.26 to 114.23) | 7.81 (6.95 to 8.7) | 85.68 (69.42 to 106.29) | 6.64 (5.23 to 8.4) | -0.16 (-0.43 to 0.1) |
| France | 1024.12 (941.92 to 1105.18) | 5.72 (5.16 to 6.32) | 1175.72 (1000.71 to 1324.25) | 6.62 (5.45 to 7.68) | 0.39 (0.35 to 0.43) |
| United States of America | 4600.96 (3638.27 to 5736.17) | 5.93 (4.62 to 7.5) | 6042.14 (5148.39 to 7114.53) | 6.55 (5.4 to 7.88) | 0.5 (0.39 to 0.62) |
| Japan | 800.67 (610.25 to 1031.43) | 1.92 (1.44 to 2.52) | 1589.62 (1262.94 to 1983.16) | 6.27 (4.91 to 7.96) | 2.48 (1.7 to 3.27) |
| Spain | 627.53 (569.58 to 692.81) | 4.58 (4.03 to 5.19) | 615.84 (553.09 to 685.7) | 6.21 (5.46 to 7.08) | 0.82 (0.68 to 0.96) |
| Croatia | 71.64 (62.81 to 80.65) | 4.92 (4.21 to 5.7) | 52.15 (44.44 to 60.62) | 5.72 (4.74 to 6.8) | 0.71 (0.62 to 0.8) |
| United Kingdom | 725.27 (590.37 to 891.43) | 4.42 (3.52 to 5.53) | 851.01 (690.96 to 1042.7) | 5.24 (4.17 to 6.54) | 0.52 (0.32 to 0.72) |
| Australia | 145.12 (112.91 to 181.77) | 2.53 (1.93 to 3.21) | 309.14 (252.03 to 378.58) | 4.93 (3.93 to 6.17) | 1.85 (1.39 to 2.32) |
| Italy | 828.87 (665.68 to 1026.29) | 4.72 (3.72 to 5.93) | 604.46 (490.34 to 741.6) | 4.89 (3.87 to 6.12) | 0.05 (-0.09 to 0.2) |
| Slovenia | 23.79 (19.31 to 29.49) | 3.84 (3.04 to 4.84) | 19.28 (15.69 to 23.64) | 4.71 (3.72 to 5.88) | 0.43 (0.27 to 0.6) |
| Poland | 502.63 (389.44 to 646.01) | 3.9 (2.95 to 5.09) | 377.79 (310.44 to 462.38) | 4.71 (3.78 to 5.83) | 0.64 (0.52 to 0.76) |
| Sweden | 104.36 (82.67 to 130.75) | 4.48 (3.47 to 5.71) | 109.88 (87.41 to 136.88) | 4.44 (3.43 to 5.64) | -0.33 (-0.47 to -0.2) |
| Germany | 565.16 (447.68 to 734.01) | 3.04 (2.34 to 4.03) | 761.18 (606.18 to 948.24) | 4.38 (3.41 to 5.58) | 0.61 (0.35 to 0.87) |
| New Zealand | 39.43 (31.26 to 49.76) | 3.16 (2.47 to 4.02) | 54.05 (44.18 to 66.11) | 4.25 (3.41 to 5.32) | 1.21 (0.91 to 1.51) |
| Greenland | 0.57 (0.43 to 0.74) | 3.5 (2.62 to 4.6) | 0.66 (0.5 to 0.84) | 4.18 (3.15 to 5.49) | 0.51 (0.41 to 0.62) |
| Slovakia | 77.98 (63.56 to 94.76) | 4.12 (3.25 to 5.09) | 48.09 (38.8 to 58.56) | 4.16 (3.26 to 5.16) | 0.05 (-0.12 to 0.21) |
| Iceland | 2.37 (1.87 to 3.02) | 2.64 (2.04 to 3.39) | 3.84 (3.03 to 4.88) | 4.09 (3.15 to 5.27) | 0.67 (0.26 to 1.09) |
| Switzerland | 61.46 (49.16 to 77.53) | 3.52 (2.78 to 4.53) | 75.15 (61.28 to 93.77) | 4.08 (3.22 to 5.17) | 0.29 (0.06 to 0.52) |
| Luxembourg | 3.45 (2.7 to 4.3) | 3.66 (2.8 to 4.65) | 5.48 (4.34 to 6.88) | 3.82 (2.96 to 4.91) | 0.15 (0.04 to 0.26) |
| Czechia | 60.85 (48.62 to 76.68) | 1.72 (1.34 to 2.21) | 77.27 (64.13 to 92.88) | 3.56 (2.89 to 4.38) | 1.75 (1.5 to 2.01) |
| Serbia | 76.63 (58.84 to 98.03) | 2.5 (1.87 to 3.24) | 75.83 (60.18 to 95.58) | 3.4 (2.64 to 4.36) | 0.58 (0.38 to 0.77) |
| Belgium | 94.16 (82.6 to 107.34) | 3.43 (2.93 to 4.02) | 89.82 (70.18 to 114.83) | 3.29 (2.51 to 4.25) | -0.44 (-0.67 to -0.22) |
| Republic of Korea | 210.62 (170.75 to 259.02) | 1.13 (0.89 to 1.42) | 332.82 (299.81 to 369.59) | 3.08 (2.71 to 3.5) | 2.16 (1.6 to 2.73) |
| Bulgaria | 65.46 (50.5 to 83.75) | 2.46 (1.87 to 3.21) | 37.64 (28.93 to 48.18) | 2.71 (2.05 to 3.52) | 0.26 (0.2 to 0.32) |
| Bosnia and Herzegovina | 36.56 (29.3 to 45.94) | 2.22 (1.74 to 2.86) | 20.59 (16.65 to 25.28) | 2.69 (2.12 to 3.36) | 0.73 (0.65 to 0.81) |
| North Macedonia | 19.17 (14.84 to 24.37) | 2.57 (1.94 to 3.33) | 13.55 (10.52 to 17.27) | 2.65 (2.01 to 3.44) | 0.16 (0.14 to 0.19) |
| Montenegro | 5.99 (4.64 to 7.66) | 2.63 (1.99 to 3.43) | 4.49 (3.48 to 5.68) | 2.65 (2.01 to 3.41) | 0.13 (0.09 to 0.18) |
| Lithuania | 19.47 (15.67 to 24.45) | 1.67 (1.29 to 2.13) | 15.28 (12.98 to 17.93) | 2.6 (2.15 to 3.14) | 1.14 (0.91 to 1.36) |
| Albania | 35.07 (27.21 to 44.9) | 2.4 (1.83 to 3.13) | 19.88 (15.16 to 25.37) | 2.59 (1.95 to 3.35) | 0.35 (0.31 to 0.38) |
| Malta | 2.64 (2.03 to 3.38) | 2.16 (1.62 to 2.87) | 2.23 (1.77 to 2.86) | 2.54 (1.95 to 3.28) | 0.36 (0.25 to 0.47) |
| Ireland | 36.52 (28.89 to 45.91) | 2.46 (1.9 to 3.17) | 35.39 (28.02 to 44.5) | 2.52 (1.94 to 3.22) | -0.19 (-0.36 to -0.03) |
| Portugal | 48 (38.08 to 60.97) | 1.33 (1.02 to 1.73) | 59.07 (48.11 to 71.93) | 2.47 (1.95 to 3.08) | 1.67 (1.27 to 2.07) |
| Greece | 62.08 (49.36 to 78) | 1.87 (1.44 to 2.4) | 51.29 (42.23 to 61.54) | 2.33 (1.88 to 2.87) | 0.42 (0.25 to 0.6) |
| Latvia | 11.93 (9.33 to 14.98) | 1.54 (1.18 to 1.97) | 8.37 (6.6 to 10.73) | 2.12 (1.63 to 2.78) | 0.61 (0.41 to 0.82) |
| Monaco | 0.11 (0.09 to 0.14) | 2.04 (1.53 to 2.7) | 0.16 (0.12 to 0.21) | 2.1 (1.58 to 2.77) | 0.1 (0.07 to 0.12) |
| Romania | 146.1 (112.11 to 188.89) | 1.76 (1.31 to 2.3) | 93.18 (74.85 to 115.06) | 2.09 (1.64 to 2.62) | 0.61 (0.52 to 0.7) |
| Andorra | 0.32 (0.24 to 0.41) | 1.94 (1.47 to 2.55) | 0.37 (0.28 to 0.48) | 2.06 (1.56 to 2.69) | 0.19 (0.14 to 0.24) |
| San Marino | 0.15 (0.12 to 0.19) | 1.92 (1.45 to 2.49) | 0.18 (0.14 to 0.23) | 2.05 (1.53 to 2.69) | 0.19 (0.15 to 0.22) |
| Israel | 37.04 (29.63 to 46.02) | 1.79 (1.4 to 2.28) | 61.51 (50.5 to 75.18) | 1.92 (1.53 to 2.39) | -0.34 (-0.7 to 0.01) |
| Cyprus | 2.81 (2.17 to 3.59) | 1.05 (0.78 to 1.37) | 5.38 (4.43 to 6.53) | 1.89 (1.51 to 2.35) | 1.47 (1.04 to 1.91) |
| Jordan | 24.44 (19.42 to 30.46) | 1.18 (0.91 to 1.51) | 81.48 (63.06 to 105.17) | 1.57 (1.18 to 2.06) | 1.01 (0.8 to 1.22) |
| Turkey | 302.1 (241.72 to 373.44) | 1.07 (0.82 to 1.35) | 415.33 (323.21 to 523.72) | 1.57 (1.19 to 2.01) | 1.06 (0.86 to 1.27) |
| Netherlands | 226.2 (201.61 to 253.36) | 5.12 (4.45 to 5.92) | 63.91 (49.94 to 81) | 1.49 (1.14 to 1.93) | -3.79 (-4.8 to -2.77) |
| Estonia | 5.5 (4.44 to 6.85) | 1.16 (0.9 to 1.47) | 4.04 (3.12 to 5.12) | 1.45 (1.09 to 1.89) | 0.67 (0.6 to 0.73) |
| Brunei Darussalam | 1.45 (1.08 to 1.91) | 1.34 (0.98 to 1.82) | 2.1 (1.6 to 2.8) | 1.41 (1.04 to 1.91) | 0.16 (0.15 to 0.18) |
| Republic of Moldova | 14.82 (11.32 to 19.44) | 0.96 (0.71 to 1.27) | 10.85 (8.88 to 13.13) | 1.28 (1.02 to 1.6) | 0.84 (0.77 to 0.9) |
| Belarus | 35 (26.97 to 44.97) | 1.09 (0.82 to 1.42) | 24.79 (19.08 to 31.81) | 1.23 (0.92 to 1.61) | 0.4 (0.38 to 0.43) |
| Kazakhstan | 63.06 (47.33 to 82.22) | 0.97 (0.72 to 1.29) | 59.31 (45.51 to 76.35) | 1.06 (0.78 to 1.4) | 0.2 (0.16 to 0.24) |
| Turkmenistan | 16.88 (12.88 to 21.87) | 0.98 (0.72 to 1.29) | 19.18 (14.68 to 24.63) | 1.06 (0.79 to 1.4) | 0.34 (0.31 to 0.38) |
| Singapore | 11.79 (8.91 to 15.36) | 1.08 (0.8 to 1.44) | 11.08 (8.44 to 14.56) | 1.05 (0.77 to 1.43) | -0.18 (-0.28 to -0.08) |
| Azerbaijan | 30.36 (23.24 to 39.48) | 0.99 (0.74 to 1.32) | 30.93 (23.65 to 39.9) | 1.03 (0.77 to 1.36) | 0.18 (0.15 to 0.21) |
| Mongolia | 9.87 (7.51 to 12.78) | 0.93 (0.69 to 1.23) | 10.62 (8.01 to 13.68) | 1.02 (0.75 to 1.35) | 0.32 (0.3 to 0.33) |
| Georgia | 18.61 (14.26 to 23.92) | 1 (0.75 to 1.32) | 8.92 (6.66 to 11.69) | 1 (0.74 to 1.33) | -0.03 (-0.06 to 0) |
| Uzbekistan | 86.03 (65.45 to 111.73) | 0.89 (0.66 to 1.18) | 127.67 (96.78 to 166.26) | 1 (0.75 to 1.33) | 0.45 (0.42 to 0.47) |
| Armenia | 11.46 (8.71 to 14.71) | 0.9 (0.66 to 1.2) | 7.56 (5.78 to 9.75) | 1 (0.75 to 1.32) | 0.38 (0.36 to 0.39) |
| Russian Federation | 436.68 (334.29 to 561.32) | 0.95 (0.71 to 1.24) | 328.92 (251.92 to 423.43) | 0.98 (0.73 to 1.29) | 0.05 (-0.02 to 0.12) |
| Kyrgyzstan | 18.28 (13.99 to 23.7) | 0.93 (0.69 to 1.24) | 22.88 (17.59 to 29.48) | 0.95 (0.7 to 1.26) | 0.02 (0 to 0.04) |
| Tajikistan | 22.57 (17.33 to 29.18) | 0.89 (0.66 to 1.19) | 36.06 (27.44 to 47.19) | 0.93 (0.69 to 1.23) | 0.09 (0.06 to 0.13) |
| Brazil | 720.55 (573.05 to 900.53) | 1.05 (0.81 to 1.34) | 627.82 (494.73 to 792.96) | 0.9 (0.69 to 1.16) | -0.37 (-0.44 to -0.3) |
| Barbados | 0.77 (0.58 to 1) | 0.81 (0.59 to 1.09) | 0.69 (0.52 to 0.9) | 0.83 (0.61 to 1.12) | 0.04 (0.01 to 0.07) |
| Kuwait | 7.69 (6.6 to 8.9) | 1.22 (1.01 to 1.44) | 8.47 (6.36 to 11.2) | 0.79 (0.58 to 1.08) | -1.55 (-1.97 to -1.12) |
| Egypt | 219.18 (170.38 to 276.7) | 0.85 (0.63 to 1.1) | 321.92 (236.46 to 441.71) | 0.77 (0.55 to 1.08) | -0.91 (-1.23 to -0.6) |
| Ukraine | 113.77 (85.47 to 148.66) | 0.72 (0.53 to 0.96) | 69.89 (53.29 to 90.55) | 0.76 (0.56 to 1) | 0.14 (0.12 to 0.17) |
| Chile | 37.44 (29.07 to 48.54) | 0.69 (0.52 to 0.91) | 39.18 (30.37 to 50.87) | 0.75 (0.56 to 0.99) | 0.48 (-0.02 to 0.99) |
| United Arab Emirates | 3.74 (2.78 to 4.93) | 0.65 (0.47 to 0.87) | 11.8 (9.02 to 15.58) | 0.75 (0.55 to 1.02) | 0.5 (0.4 to 0.61) |
| Algeria | 91.81 (70.92 to 116.9) | 0.71 (0.53 to 0.93) | 101.85 (76.4 to 136) | 0.73 (0.53 to 1.01) | -0.24 (-0.43 to -0.05) |
| Qatar | 0.81 (0.6 to 1.1) | 0.61 (0.44 to 0.85) | 3.48 (2.65 to 4.61) | 0.71 (0.52 to 0.97) | 0.57 (0.55 to 0.6) |
| Lebanon | 7.31 (5.44 to 9.89) | 0.51 (0.36 to 0.71) | 10.92 (8.04 to 14.54) | 0.69 (0.49 to 0.95) | 1.05 (0.97 to 1.13) |
| China | 1675.81 (1223.36 to 2243.34) | 0.34 (0.24 to 0.46) | 2140.47 (1633.94 to 2740.23) | 0.68 (0.51 to 0.89) | 2.67 (2.29 to 3.05) |
| Bahrain | 1.2 (0.92 to 1.52) | 0.68 (0.51 to 0.89) | 2.42 (1.85 to 3.22) | 0.67 (0.49 to 0.92) | -0.42 (-0.63 to -0.22) |
| Puerto Rico | 10.86 (9.07 to 13.15) | 0.75 (0.61 to 0.94) | 6.3 (5.08 to 7.78) | 0.67 (0.52 to 0.86) | -0.14 (-0.25 to -0.02) |
| Saudi Arabia | 48.4 (37.95 to 61.73) | 0.63 (0.48 to 0.84) | 71.36 (53.62 to 92.36) | 0.66 (0.49 to 0.88) | 0.23 (0.08 to 0.39) |
| Syrian Arab Republic | 38.13 (28.38 to 50.88) | 0.56 (0.4 to 0.77) | 46.86 (34.79 to 62.86) | 0.64 (0.46 to 0.88) | 0.32 (0.25 to 0.38) |
| Tunisia | 21.75 (16.28 to 29.31) | 0.55 (0.4 to 0.76) | 21.84 (16.19 to 29.23) | 0.61 (0.44 to 0.85) | 0.43 (0.4 to 0.46) |
| Palestine | 5.09 (3.82 to 6.75) | 0.48 (0.35 to 0.67) | 14.32 (10.57 to 19.48) | 0.61 (0.43 to 0.84) | 0.72 (0.65 to 0.8) |
| Taiwan (Province of China) | 20.55 (15.51 to 26.49) | 0.26 (0.19 to 0.34) | 29.33 (23.32 to 36.35) | 0.6 (0.46 to 0.76) | 3.8 (3.33 to 4.28) |
| Oman | 4.54 (3.4 to 6.07) | 0.55 (0.39 to 0.75) | 6.93 (5.13 to 9.17) | 0.59 (0.43 to 0.81) | 0.1 (-0.01 to 0.21) |
| Sudan | 48.88 (36.14 to 66.55) | 0.49 (0.35 to 0.69) | 116.73 (86.96 to 154.42) | 0.58 (0.42 to 0.79) | 0.55 (0.54 to 0.57) |
| Paraguay | 12.82 (9.77 to 16.52) | 0.68 (0.5 to 0.9) | 15.51 (11.42 to 20.54) | 0.56 (0.4 to 0.76) | -1.09 (-1.36 to -0.82) |
| Iraq | 42.06 (31.74 to 55.79) | 0.47 (0.34 to 0.63) | 106.09 (79.56 to 144.57) | 0.56 (0.41 to 0.78) | 0.62 (0.6 to 0.65) |
| Yemen | 32.52 (24.28 to 43.7) | 0.47 (0.34 to 0.65) | 85.1 (62.23 to 113.5) | 0.54 (0.39 to 0.75) | 0.57 (0.53 to 0.61) |
| Bahamas | 0.53 (0.39 to 0.7) | 0.46 (0.33 to 0.63) | 0.68 (0.49 to 0.92) | 0.52 (0.37 to 0.73) | 0.39 (0.34 to 0.43) |
| Trinidad and Tobago | 2.39 (1.78 to 3.23) | 0.47 (0.33 to 0.64) | 1.97 (1.47 to 2.66) | 0.51 (0.36 to 0.7) | 0.17 (0.12 to 0.22) |
| Libya | 8.52 (6.3 to 11.74) | 0.38 (0.27 to 0.54) | 12.34 (9.1 to 16.65) | 0.51 (0.37 to 0.71) | 0.77 (0.58 to 0.95) |
| Afghanistan | 29.3 (21.38 to 40.11) | 0.48 (0.34 to 0.68) | 101.33 (74.73 to 139.34) | 0.51 (0.36 to 0.71) | 0.23 (0.16 to 0.3) |
| United States Virgin Islands | 0.19 (0.15 to 0.26) | 0.45 (0.33 to 0.62) | 0.14 (0.11 to 0.19) | 0.51 (0.36 to 0.69) | 0.35 (0.3 to 0.4) |
| Dominica | 0.14 (0.11 to 0.19) | 0.43 (0.3 to 0.58) | 0.12 (0.09 to 0.15) | 0.49 (0.35 to 0.66) | 0.43 (0.38 to 0.48) |
| Bermuda | 0.08 (0.06 to 0.1) | 0.45 (0.33 to 0.62) | 0.07 (0.05 to 0.09) | 0.49 (0.36 to 0.67) | 0.22 (0.18 to 0.26) |
| Saint Kitts and Nevis | 0.08 (0.06 to 0.11) | 0.44 (0.31 to 0.61) | 0.09 (0.07 to 0.12) | 0.49 (0.35 to 0.67) | 0.26 (0.2 to 0.32) |
| Iran (Islamic Republic of) | 142.81 (103.08 to 197.39) | 0.49 (0.34 to 0.7) | 120.55 (87.66 to 167.04) | 0.48 (0.34 to 0.68) | -0.16 (-0.39 to 0.07) |
| Grenada | 0.17 (0.12 to 0.22) | 0.42 (0.29 to 0.58) | 0.17 (0.12 to 0.22) | 0.47 (0.33 to 0.65) | 0.31 (0.26 to 0.37) |
| Mexico | 214.72 (160.8 to 279.56) | 0.49 (0.36 to 0.65) | 220.56 (167.51 to 285.08) | 0.47 (0.35 to 0.63) | -0.08 (-0.15 to 0) |
| Guyana | 1.6 (1.17 to 2.13) | 0.42 (0.3 to 0.58) | 1.4 (1.02 to 1.84) | 0.46 (0.33 to 0.63) | 0.22 (0.17 to 0.26) |
| Antigua and Barbuda | 0.11 (0.08 to 0.14) | 0.43 (0.31 to 0.58) | 0.13 (0.09 to 0.17) | 0.46 (0.33 to 0.63) | 0.2 (0.16 to 0.25) |
| Suriname | 0.74 (0.55 to 0.97) | 0.43 (0.3 to 0.58) | 0.94 (0.69 to 1.25) | 0.46 (0.33 to 0.63) | 0.23 (0.19 to 0.27) |
| Saint Lucia | 0.29 (0.21 to 0.39) | 0.43 (0.3 to 0.59) | 0.25 (0.18 to 0.34) | 0.46 (0.33 to 0.65) | 0.15 (0.09 to 0.21) |
| Jamaica | 4.52 (3.31 to 5.97) | 0.4 (0.29 to 0.55) | 4.48 (3.31 to 5.95) | 0.45 (0.32 to 0.61) | 0.3 (0.25 to 0.36) |
| Dominican Republic | 13.86 (10.2 to 18.73) | 0.4 (0.28 to 0.55) | 18.42 (13.47 to 24.66) | 0.45 (0.32 to 0.62) | 0.42 (0.38 to 0.46) |
| Saint Vincent and the Grenadines | 0.23 (0.17 to 0.3) | 0.41 (0.29 to 0.56) | 0.17 (0.13 to 0.23) | 0.45 (0.32 to 0.61) | 0.22 (0.16 to 0.28) |
| Cuba | 18.12 (13.46 to 24.37) | 0.42 (0.3 to 0.59) | 12.35 (9.05 to 16.46) | 0.44 (0.32 to 0.61) | 0.08 (0.01 to 0.14) |
| Belize | 0.38 (0.28 to 0.51) | 0.39 (0.28 to 0.55) | 0.83 (0.61 to 1.11) | 0.44 (0.32 to 0.61) | 0.3 (0.26 to 0.35) |
| Viet Nam | 78.22 (53.27 to 107.53) | 0.24 (0.16 to 0.34) | 119.4 (85.47 to 161.19) | 0.41 (0.28 to 0.56) | 1.93 (1.53 to 2.32) |
| Uruguay | 3.97 (2.88 to 5.2) | 0.35 (0.25 to 0.48) | 4.27 (3.12 to 5.61) | 0.41 (0.29 to 0.56) | 0.39 (0.33 to 0.44) |
| El Salvador | 11.08 (8.12 to 14.78) | 0.41 (0.29 to 0.56) | 10.02 (7.37 to 13.5) | 0.41 (0.29 to 0.57) | -0.02 (-0.07 to 0.03) |
| Panama | 5.23 (3.98 to 6.69) | 0.47 (0.35 to 0.62) | 6.32 (4.62 to 8.45) | 0.41 (0.29 to 0.56) | -0.75 (-0.93 to -0.58) |
| Venezuela (Bolivarian Republic of) | 41 (30.8 to 52.98) | 0.46 (0.34 to 0.62) | 39.76 (29.36 to 52.83) | 0.41 (0.29 to 0.55) | -0.84 (-1.04 to -0.64) |
| Colombia | 61.21 (45.07 to 81.37) | 0.41 (0.29 to 0.56) | 66.88 (49.33 to 89.78) | 0.41 (0.3 to 0.56) | -0.06 (-0.16 to 0.04) |
| Costa Rica | 5.87 (4.43 to 7.74) | 0.44 (0.32 to 0.59) | 6.28 (4.68 to 8.25) | 0.41 (0.3 to 0.55) | -0.29 (-0.37 to -0.21) |
| Argentina | 49.96 (36.34 to 66.32) | 0.38 (0.27 to 0.52) | 59.83 (43.62 to 79.21) | 0.4 (0.28 to 0.54) | 0.06 (0.04 to 0.07) |
| Haiti | 10.99 (8.11 to 14.5) | 0.37 (0.26 to 0.5) | 21.55 (15.74 to 29.34) | 0.4 (0.28 to 0.56) | 0.19 (0.16 to 0.21) |
| Morocco | 44.32 (33.03 to 58.11) | 0.37 (0.26 to 0.49) | 53.61 (39.63 to 71.12) | 0.4 (0.29 to 0.55) | 0.28 (0.25 to 0.32) |
| Guatemala | 14.94 (11.07 to 20.04) | 0.38 (0.27 to 0.52) | 31.94 (23.56 to 42.77) | 0.39 (0.28 to 0.54) | 0.05 (0 to 0.1) |
| Sri Lanka | 15.29 (10.82 to 20.65) | 0.2 (0.14 to 0.28) | 27.89 (21.56 to 35.56) | 0.37 (0.28 to 0.49) | 2.06 (1.71 to 2.41) |
| Nicaragua | 7.94 (5.72 to 10.53) | 0.39 (0.27 to 0.53) | 10.19 (7.37 to 13.31) | 0.37 (0.26 to 0.5) | -0.15 (-0.2 to -0.1) |
| Honduras | 9.38 (6.95 to 12.67) | 0.39 (0.28 to 0.54) | 16.98 (12.32 to 22.92) | 0.37 (0.27 to 0.51) | -0.17 (-0.24 to -0.1) |
| Ecuador | 13.49 (10.23 to 17.64) | 0.27 (0.2 to 0.37) | 25.42 (19.88 to 32.02) | 0.36 (0.27 to 0.48) | 0.56 (0.36 to 0.76) |
| Democratic People's Republic of Korea | 23.97 (17.49 to 32.31) | 0.31 (0.21 to 0.42) | 25.64 (18.67 to 34.27) | 0.35 (0.24 to 0.48) | 0.53 (0.47 to 0.58) |
| Bangladesh | 153.17 (109.73 to 213.8) | 0.28 (0.19 to 0.41) | 213.83 (152.24 to 301.89) | 0.33 (0.23 to 0.47) | 0.43 (0.39 to 0.47) |
| Nepal | 24.74 (17.57 to 34.98) | 0.27 (0.18 to 0.39) | 44.68 (31.05 to 62.09) | 0.32 (0.22 to 0.46) | 0.61 (0.58 to 0.64) |
| Bhutan | 0.88 (0.61 to 1.25) | 0.27 (0.19 to 0.4) | 0.89 (0.62 to 1.26) | 0.3 (0.2 to 0.44) | 0.27 (0.22 to 0.32) |
| India | 1203.02 (852.86 to 1685.94) | 0.31 (0.21 to 0.45) | 1599.45 (1131.06 to 2224.24) | 0.28 (0.19 to 0.4) | -0.08 (-0.3 to 0.14) |
| Pakistan | 139.34 (98.71 to 195.21) | 0.25 (0.17 to 0.36) | 305.61 (215.89 to 431.11) | 0.28 (0.19 to 0.4) | 0.38 (0.36 to 0.4) |
| Peru | 28.14 (19.9 to 38.19) | 0.27 (0.18 to 0.37) | 33.49 (24.19 to 45.67) | 0.27 (0.19 to 0.38) | -0.08 (-0.19 to 0.02) |
| Malaysia | 13.26 (9.73 to 17.48) | 0.16 (0.12 to 0.22) | 29.49 (22.75 to 37.73) | 0.26 (0.19 to 0.35) | 1.4 (1.21 to 1.59) |
| Bolivia (Plurinational State of) | 7.91 (5.66 to 10.89) | 0.26 (0.17 to 0.36) | 12.27 (8.83 to 16.51) | 0.26 (0.18 to 0.36) | -0.15 (-0.24 to -0.07) |
| Equatorial Guinea | 0.35 (0.24 to 0.5) | 0.17 (0.11 to 0.25) | 1.95 (1.4 to 2.64) | 0.25 (0.17 to 0.35) | 1.44 (1.36 to 1.52) |
| Gabon | 0.97 (0.67 to 1.32) | 0.21 (0.14 to 0.29) | 1.87 (1.28 to 2.56) | 0.24 (0.16 to 0.34) | 0.52 (0.49 to 0.54) |
| Botswana | 1.48 (1.02 to 2.07) | 0.21 (0.14 to 0.3) | 2.21 (1.54 to 3.06) | 0.24 (0.16 to 0.33) | 0.42 (0.39 to 0.44) |
| Congo | 2.51 (1.75 to 3.44) | 0.2 (0.13 to 0.28) | 5.44 (3.84 to 7.52) | 0.23 (0.15 to 0.32) | 0.42 (0.38 to 0.46) |
| Eswatini | 0.9 (0.62 to 1.27) | 0.21 (0.14 to 0.3) | 1.23 (0.86 to 1.69) | 0.23 (0.15 to 0.32) | 0.31 (0.3 to 0.33) |
| Namibia | 1.51 (1.04 to 2.14) | 0.21 (0.14 to 0.3) | 2.4 (1.68 to 3.31) | 0.22 (0.15 to 0.32) | 0.28 (0.24 to 0.31) |
| Angola | 9.2 (6.32 to 12.87) | 0.18 (0.12 to 0.26) | 32 (22.19 to 44.35) | 0.21 (0.14 to 0.3) | 0.46 (0.43 to 0.48) |
| Ghana | 13.97 (9.58 to 19.09) | 0.19 (0.12 to 0.27) | 30.95 (21.7 to 42.52) | 0.21 (0.14 to 0.3) | 0.28 (0.22 to 0.34) |
| Cameroon | 10.13 (7 to 14.14) | 0.2 (0.13 to 0.28) | 31.28 (21.66 to 43.42) | 0.21 (0.14 to 0.3) | 0.09 (0.02 to 0.17) |
| Zimbabwe | 11.48 (8.02 to 16.02) | 0.2 (0.13 to 0.29) | 15.41 (10.71 to 21.57) | 0.21 (0.14 to 0.3) | -0.12 (-0.18 to -0.05) |
| Lesotho | 1.78 (1.22 to 2.48) | 0.19 (0.13 to 0.28) | 1.99 (1.39 to 2.78) | 0.21 (0.14 to 0.31) | 0.31 (0.29 to 0.33) |
| Cabo Verde | 0.32 (0.22 to 0.44) | 0.18 (0.12 to 0.25) | 0.45 (0.32 to 0.61) | 0.21 (0.14 to 0.3) | 0.41 (0.38 to 0.44) |
| South Africa | 33.78 (23.6 to 46.18) | 0.19 (0.13 to 0.27) | 39.9 (28.14 to 54.73) | 0.2 (0.13 to 0.28) | 0.16 (0.13 to 0.18) |
| Gambia | 0.82 (0.55 to 1.13) | 0.17 (0.11 to 0.24) | 2.29 (1.56 to 3.23) | 0.2 (0.13 to 0.28) | 0.56 (0.55 to 0.58) |
| Togo | 3.4 (2.37 to 4.71) | 0.18 (0.12 to 0.26) | 7.56 (5.19 to 10.58) | 0.2 (0.13 to 0.29) | 0.22 (0.15 to 0.29) |
| Sao Tome and Principe | 0.11 (0.08 to 0.16) | 0.18 (0.11 to 0.25) | 0.2 (0.15 to 0.28) | 0.2 (0.14 to 0.28) | 0.39 (0.32 to 0.47) |
| Senegal | 7.03 (4.83 to 9.91) | 0.18 (0.12 to 0.26) | 14.84 (10.37 to 20.66) | 0.2 (0.13 to 0.28) | 0.13 (0.07 to 0.19) |
| Mauritania | 1.93 (1.32 to 2.68) | 0.19 (0.13 to 0.28) | 4.12 (2.89 to 5.59) | 0.2 (0.13 to 0.28) | 0.09 (0.01 to 0.16) |
| Zambia | 8.14 (5.6 to 11.27) | 0.19 (0.13 to 0.27) | 18.79 (13 to 25.86) | 0.2 (0.13 to 0.28) | 0.31 (0.27 to 0.35) |
| Rwanda | 6.36 (4.28 to 8.94) | 0.18 (0.11 to 0.26) | 11.72 (8.04 to 16.18) | 0.19 (0.12 to 0.27) | 0.29 (0.24 to 0.34) |
| Djibouti | 0.44 (0.31 to 0.62) | 0.18 (0.12 to 0.26) | 0.91 (0.63 to 1.25) | 0.19 (0.12 to 0.27) | 0.12 (0.05 to 0.18) |
| Seychelles | 0.05 (0.04 to 0.07) | 0.16 (0.1 to 0.23) | 0.06 (0.04 to 0.08) | 0.19 (0.13 to 0.27) | 0.44 (0.4 to 0.49) |
| Mauritius | 0.7 (0.47 to 0.97) | 0.15 (0.1 to 0.22) | 0.68 (0.48 to 0.93) | 0.19 (0.13 to 0.26) | 0.62 (0.59 to 0.64) |
| Eritrea | 2.61 (1.77 to 3.6) | 0.17 (0.11 to 0.24) | 6.34 (4.3 to 8.78) | 0.19 (0.12 to 0.27) | 0.37 (0.32 to 0.41) |
| Democratic Republic of the Congo | 34.19 (23.21 to 47.34) | 0.18 (0.12 to 0.26) | 86.8 (59.22 to 124.12) | 0.19 (0.13 to 0.28) | 0.18 (0.1 to 0.26) |
| Madagascar | 10.83 (7.51 to 15.06) | 0.18 (0.12 to 0.26) | 24.82 (17.3 to 34.36) | 0.19 (0.12 to 0.26) | 0.17 (0.16 to 0.19) |
| Benin | 4.23 (2.95 to 5.94) | 0.18 (0.12 to 0.26) | 12.27 (8.57 to 16.99) | 0.19 (0.13 to 0.28) | 0.14 (0.09 to 0.2) |
| Comoros | 0.43 (0.3 to 0.6) | 0.18 (0.12 to 0.25) | 0.61 (0.42 to 0.84) | 0.19 (0.13 to 0.27) | 0.2 (0.16 to 0.24) |
| United Republic of Tanzania | 23.24 (16.09 to 32.25) | 0.17 (0.11 to 0.25) | 53.25 (36.71 to 73.76) | 0.19 (0.12 to 0.27) | 0.3 (0.24 to 0.36) |
| Guinea-Bissau | 0.9 (0.62 to 1.25) | 0.18 (0.11 to 0.25) | 1.8 (1.24 to 2.53) | 0.19 (0.12 to 0.27) | 0.16 (0.12 to 0.21) |
| Sierra Leone | 2.98 (2.05 to 4.21) | 0.18 (0.12 to 0.26) | 7.77 (5.35 to 10.76) | 0.19 (0.12 to 0.27) | 0.06 (-0.02 to 0.14) |
| Uganda | 14.66 (9.87 to 20.61) | 0.16 (0.11 to 0.24) | 42.02 (29.16 to 57.7) | 0.19 (0.12 to 0.27) | 0.53 (0.49 to 0.57) |
| Liberia | 1.79 (1.25 to 2.52) | 0.18 (0.12 to 0.26) | 4.63 (3.2 to 6.39) | 0.19 (0.13 to 0.28) | 0.24 (0.14 to 0.34) |
| Malawi | 8.13 (5.47 to 11.49) | 0.17 (0.11 to 0.25) | 19.02 (12.99 to 26.58) | 0.18 (0.12 to 0.26) | 0.3 (0.27 to 0.32) |
| Kenya | 19.45 (13.35 to 27.08) | 0.16 (0.1 to 0.22) | 45.11 (31.35 to 62.15) | 0.18 (0.12 to 0.25) | 0.27 (0.21 to 0.33) |
| Guinea | 4.85 (3.22 to 6.87) | 0.17 (0.11 to 0.25) | 11.5 (7.93 to 15.83) | 0.18 (0.12 to 0.25) | 0.1 (0.07 to 0.12) |
| Central African Republic | 2.4 (1.64 to 3.36) | 0.18 (0.12 to 0.26) | 4.75 (3.22 to 6.61) | 0.18 (0.12 to 0.26) | -0.05 (-0.07 to -0.03) |
| Guam | 0.08 (0.06 to 0.11) | 0.15 (0.1 to 0.22) | 0.11 (0.08 to 0.15) | 0.18 (0.12 to 0.26) | 0.68 (0.64 to 0.72) |
| Nigeria | 70.28 (49.01 to 98.03) | 0.16 (0.11 to 0.23) | 199.07 (139.13 to 274.81) | 0.18 (0.12 to 0.25) | 0.23 (0.15 to 0.31) |
| Northern Mariana Islands | 0.03 (0.02 to 0.03) | 0.15 (0.1 to 0.22) | 0.03 (0.02 to 0.04) | 0.18 (0.12 to 0.25) | 0.41 (0.37 to 0.45) |
| Cook Islands | 0.01 (0.01 to 0.02) | 0.14 (0.09 to 0.21) | 0.01 (0.01 to 0.01) | 0.18 (0.12 to 0.25) | 0.66 (0.6 to 0.72) |
| Palau | 0.01 (0.01 to 0.01) | 0.15 (0.1 to 0.21) | 0.01 (0.01 to 0.01) | 0.18 (0.12 to 0.25) | 0.57 (0.52 to 0.61) |
| South Sudan | 5.33 (3.61 to 7.4) | 0.17 (0.11 to 0.25) | 9.14 (6.39 to 12.61) | 0.18 (0.12 to 0.26) | 0.11 (0.09 to 0.13) |
| Burkina Faso | 8.11 (5.39 to 11.48) | 0.17 (0.11 to 0.24) | 20.08 (13.54 to 28.18) | 0.18 (0.11 to 0.25) | 0.11 (0.06 to 0.17) |
| Myanmar | 25.82 (17.01 to 36.42) | 0.13 (0.09 to 0.2) | 35.98 (24.32 to 49.53) | 0.17 (0.11 to 0.25) | 0.86 (0.81 to 0.92) |
| Fiji | 0.5 (0.34 to 0.69) | 0.14 (0.09 to 0.2) | 0.57 (0.4 to 0.81) | 0.17 (0.11 to 0.24) | 0.55 (0.52 to 0.57) |
| Maldives | 0.14 (0.09 to 0.2) | 0.13 (0.08 to 0.18) | 0.22 (0.16 to 0.31) | 0.17 (0.11 to 0.23) | 0.88 (0.83 to 0.94) |
| Thailand | 30.02 (19.97 to 42.42) | 0.12 (0.07 to 0.18) | 28.92 (19.98 to 39.15) | 0.17 (0.11 to 0.24) | 1.11 (0.98 to 1.23) |
| Burundi | 4.54 (3.08 to 6.34) | 0.17 (0.11 to 0.25) | 10.22 (6.97 to 14.32) | 0.17 (0.11 to 0.25) | 0.03 (0.01 to 0.05) |
| Mozambique | 10.32 (6.95 to 14.42) | 0.16 (0.1 to 0.22) | 26.87 (17.86 to 37.94) | 0.17 (0.11 to 0.25) | 0.44 (0.42 to 0.46) |
| Mali | 6.65 (4.41 to 9.51) | 0.16 (0.1 to 0.24) | 20.03 (13.87 to 28.13) | 0.17 (0.11 to 0.25) | 0.12 (0.06 to 0.18) |
| Chad | 4.78 (3.18 to 6.8) | 0.16 (0.1 to 0.24) | 14.91 (10.11 to 21.29) | 0.17 (0.11 to 0.25) | 0.1 (0.03 to 0.17) |
| Niue | 0 (0 to 0) | 0.14 (0.09 to 0.2) | 0 (0 to 0) | 0.17 (0.11 to 0.25) | 0.74 (0.68 to 0.8) |
| Timor-Leste | 0.44 (0.3 to 0.61) | 0.13 (0.08 to 0.18) | 1.11 (0.76 to 1.54) | 0.16 (0.1 to 0.23) | 1.08 (0.97 to 1.18) |
| Tonga | 0.07 (0.04 to 0.09) | 0.13 (0.08 to 0.19) | 0.07 (0.05 to 0.1) | 0.16 (0.1 to 0.23) | 0.65 (0.57 to 0.74) |
| Lao People's Democratic Republic | 2.66 (1.77 to 3.8) | 0.13 (0.08 to 0.19) | 4.88 (3.35 to 6.7) | 0.16 (0.11 to 0.23) | 0.72 (0.66 to 0.78) |
| Micronesia (Federated States of) | 0.07 (0.05 to 0.1) | 0.13 (0.08 to 0.19) | 0.07 (0.05 to 0.1) | 0.16 (0.1 to 0.22) | 0.61 (0.58 to 0.63) |
| Philippines | 41.23 (28.26 to 56.76) | 0.13 (0.09 to 0.19) | 74.56 (52.02 to 101.91) | 0.16 (0.11 to 0.23) | 0.59 (0.52 to 0.66) |
| Cambodia | 6.53 (4.37 to 9.1) | 0.13 (0.08 to 0.19) | 10.32 (7.03 to 14.38) | 0.16 (0.1 to 0.23) | 0.74 (0.71 to 0.76) |
| Ethiopia | 37.22 (25.21 to 52.37) | 0.14 (0.09 to 0.21) | 89.45 (60.54 to 124.43) | 0.16 (0.1 to 0.23) | 0.36 (0.29 to 0.43) |
| Tuvalu | 0 (0 to 0.01) | 0.13 (0.08 to 0.19) | 0.01 (0.01 to 0.01) | 0.16 (0.1 to 0.23) | 0.61 (0.58 to 0.63) |
| American Samoa | 0.03 (0.02 to 0.04) | 0.14 (0.09 to 0.2) | 0.04 (0.03 to 0.06) | 0.16 (0.11 to 0.23) | 0.57 (0.51 to 0.63) |
| Niger | 6.27 (4.19 to 8.87) | 0.16 (0.1 to 0.23) | 19.91 (13.44 to 27.98) | 0.16 (0.1 to 0.23) | -0.06 (-0.12 to 0) |
| Tokelau | 0 (0 to 0) | 0.14 (0.09 to 0.2) | 0 (0 to 0) | 0.16 (0.11 to 0.23) | 0.44 (0.34 to 0.54) |
| Nauru | 0.01 (0 to 0.01) | 0.14 (0.09 to 0.2) | 0.01 (0.01 to 0.01) | 0.16 (0.1 to 0.23) | 0.53 (0.49 to 0.57) |
| Samoa | 0.12 (0.08 to 0.16) | 0.13 (0.08 to 0.19) | 0.16 (0.11 to 0.22) | 0.15 (0.1 to 0.21) | 0.41 (0.28 to 0.54) |
| Marshall Islands | 0.03 (0.02 to 0.04) | 0.13 (0.08 to 0.18) | 0.04 (0.02 to 0.05) | 0.15 (0.1 to 0.21) | 0.62 (0.57 to 0.68) |
| Indonesia | 107.33 (71.35 to 149.95) | 0.12 (0.08 to 0.18) | 141.94 (97.62 to 195.7) | 0.15 (0.1 to 0.21) | 0.69 (0.6 to 0.77) |
| Vanuatu | 0.09 (0.06 to 0.13) | 0.12 (0.08 to 0.18) | 0.19 (0.13 to 0.27) | 0.14 (0.09 to 0.21) | 0.5 (0.47 to 0.53) |
| Kiribati | 0.04 (0.03 to 0.06) | 0.13 (0.08 to 0.19) | 0.07 (0.05 to 0.1) | 0.14 (0.09 to 0.2) | 0.19 (0.15 to 0.23) |
| Somalia | 5.65 (3.8 to 7.9) | 0.15 (0.09 to 0.21) | 15.01 (9.97 to 21.47) | 0.14 (0.09 to 0.2) | -0.08 (-0.12 to -0.05) |
| Solomon Islands | 0.21 (0.13 to 0.3) | 0.12 (0.07 to 0.17) | 0.41 (0.27 to 0.59) | 0.13 (0.08 to 0.19) | 0.42 (0.37 to 0.47) |
| Papua New Guinea | 2.27 (1.47 to 3.2) | 0.11 (0.07 to 0.17) | 5.62 (3.7 to 7.82) | 0.13 (0.08 to 0.19) | 0.44 (0.39 to 0.5) |

**Supplementary Table 3** Prevalence of IBD among children and adolescents in 1990 and 2019 and the EAPC in different countries and territories

| Location | 1990 |  | 2019 |  | 1990-2019 |
| --- | --- | --- | --- | --- | --- |
|  | Prevalence cases NO.(95%UI) | ASR/100,000 (95% CI) | Prevalence cases NO.(95%UI) | ASR/100,000 (95% CI) | EAPC (95%CI) |
| Canada | 6017.97 (5419.05 to 6656.01) | 73.42 (64.9 to 82.27) | 5620.43 (5011.86 to 6319.31) | 63.89 (56.17 to 72.39) | -0.12  (-0.28 to 0.03) |
| Denmark | 494.77 (432.59 to 565.96) | 32.1 (27.62 to 37.3) | 494.77 (423.8 to 581.5) | 33.52 (28.45 to 39.43) | 0.41  (0.26 to 0.55) |
| Hungary | 769.67 (648.94 to 908.95) | 22.65 (18.92 to 26.77) | 674.78 (577.24 to 796.61) | 32.03 (27.02 to 38.15) | 1.65  (1.25 to 2.06) |
| Croatia | 345.72 (298.87 to 398.45) | 23.43 (20.12 to 27.17) | 226.12 (187.9 to 268.02) | 24.53 (20.17 to 29.4) | 0.4  (0.28 to 0.52) |
| United States of America | 15612.41 (12329.2 to 19593.36) | 19.92 (15.75 to 25.13) | 20883.9 (17621.78 to 24674.02) | 22.34 (18.58 to 26.77) | 0.53  (0.38 to 0.69) |
| Austria | 521.87 (428.19 to 631.53) | 23.78 (19.22 to 29.02) | 398.88 (333.6 to 483.13) | 20.55 (16.84 to 25.16) | -0.37  (-0.73 to 0) |
| Poland | 1940.84 (1507.1 to 2475.54) | 15.09 (11.66 to 19.47) | 1636.04 (1343.04 to 1995.41) | 20.35 (16.66 to 25.04) | 1.11  (0.91 to 1.31) |
| Japan | 3056.55 (2304.2 to 3958.54) | 7.12 (5.4 to 9.27) | 5116.67 (4101.92 to 6339.4) | 19.93 (15.81 to 24.84) | 2.17  (1.46 to 2.89) |
| Slovenia | 107.48 (87.57 to 132.15) | 17.16 (13.85 to 21.31) | 78.33 (63.6 to 95.78) | 19.18 (15.4 to 23.85) | 0.05  (-0.1 to 0.21) |
| Spain | 2034.29 (1795.73 to 2308.76) | 14.47 (12.65 to 16.7) | 1904.16 (1654.62 to 2191.64) | 19.12 (16.33 to 22.28) | 0.78  (0.57 to 0.99) |
| Norway | 178.8 (139.93 to 231.11) | 13.52 (10.53 to 17.51) | 262.95 (208.97 to 330.32) | 18.9 (14.83 to 23.9) | 0.25  (-0.1 to 0.59) |
| Finland | 334.14 (289.13 to 381.69) | 24.99 (21.17 to 29.05) | 241.47 (194.92 to 303.38) | 18.63 (14.87 to 23.63) | -0.59  (-0.94 to -0.24) |
| Slovakia | 336.93 (271.56 to 413.94) | 17.73 (14.24 to 21.97) | 212.81 (169.82 to 264.76) | 18.34 (14.63 to 22.7) | 0.08  (-0.08 to 0.24) |
| France | 3077.96 (2774.4 to 3440.97) | 16.81 (14.88 to 19.05) | 3106.59 (2585.03 to 3649.03) | 17.38 (14.26 to 20.61) | 0.3  (0.18 to 0.43) |
| Australia | 508.82 (392.04 to 637.17) | 8.67 (6.65 to 10.94) | 967.87 (776.81 to 1190.27) | 15.38 (12.15 to 19.12) | 1.6  (1.17 to 2.03) |
| Italy | 2807.68 (2250.85 to 3540.01) | 15.5 (12.33 to 19.57) | 1915.14 (1534.52 to 2374.93) | 15.35 (12.23 to 19.24) | -0.2  (-0.33 to -0.06) |
| Czechia | 303.71 (243.87 to 379.18) | 8.37 (6.67 to 10.5) | 328.27 (270.73 to 402.13) | 15.28 (12.47 to 18.76) | 1.43  (1.22 to 1.65) |
| United Kingdom | 2338.05 (1907 to 2892.27) | 14 (11.32 to 17.42) | 2369.19 (1942.85 to 2942.76) | 14.56 (11.72 to 18.16) | 0.01  (-0.14 to 0.17) |
| Serbia | 343.45 (261.85 to 438.65) | 11.12 (8.46 to 14.38) | 322.34 (255.4 to 410.54) | 14.2 (11.15 to 18.2) | 0.44  (0.29 to 0.6) |
| Sweden | 341.24 (265.22 to 430.17) | 14.36 (11.02 to 18.38) | 319.58 (249.2 to 401.55) | 12.9 (10.03 to 16.25) | -0.61  (-0.71 to -0.5) |
| Germany | 1720.36 (1347.7 to 2235.06) | 9.14 (7.1 to 11.94) | 2243.62 (1803.18 to 2819.63) | 12.76 (10.01 to 16.21) | 0.44  (0.13 to 0.75) |
| New Zealand | 127.92 (101.67 to 164.3) | 10.05 (7.86 to 12.88) | 159.81 (128.86 to 200.28) | 12.52 (10 to 15.74) | 0.94  (0.63 to 1.26) |
| Switzerland | 194.45 (154.31 to 246.55) | 10.94 (8.57 to 13.96) | 230.35 (183.88 to 290.56) | 12.4 (9.79 to 15.68) | 0.28  (0.09 to 0.48) |
| Greenland | 1.78 (1.34 to 2.33) | 10.95 (8.21 to 14.56) | 1.92 (1.46 to 2.51) | 12.21 (9.16 to 16.1) | 0.29  (0.22 to 0.36) |
| Iceland | 8.46 (6.59 to 10.84) | 9.29 (7.16 to 11.89) | 11.48 (9.05 to 14.69) | 12.17 (9.5 to 15.85) | 0.17  (-0.17 to 0.52) |
| Bosnia and Herzegovina | 171.65 (136.74 to 215.64) | 10.23 (8.07 to 12.98) | 95.38 (76.91 to 117.66) | 12.09 (9.7 to 15.04) | 0.6  (0.55 to 0.65) |
| Montenegro | 27.51 (21.35 to 35.31) | 11.95 (9.26 to 15.39) | 20.72 (16.11 to 26.05) | 11.97 (9.28 to 15.2) | 0.07  (0.04 to 0.11) |
| Bulgaria | 304.65 (236.23 to 389.11) | 11.25 (8.7 to 14.42) | 162.69 (125.62 to 208.8) | 11.68 (8.93 to 14.93) | 0.07  (0.05 to 0.09) |
| North Macedonia | 85.09 (65.85 to 108.89) | 11.33 (8.69 to 14.58) | 60.51 (47.31 to 77.03) | 11.6 (8.99 to 14.9) | 0.12  (0.11 to 0.14) |
| Luxembourg | 9.93 (7.76 to 12.57) | 10.42 (8.1 to 13.28) | 16.1 (12.72 to 20.6) | 11.13 (8.67 to 14.31) | 0.15  (-0.01 to 0.3) |
| Albania | 152.43 (118.36 to 196.82) | 10.42 (8.02 to 13.56) | 87.1 (67.1 to 111.33) | 10.91 (8.38 to 14.01) | 0.22  (0.2 to 0.25) |
| Lithuania | 88 (70.04 to 110.07) | 7.41 (5.84 to 9.41) | 61.87 (51.29 to 74.97) | 10.41 (8.56 to 12.72) | 0.88  (0.7 to 1.06) |
| Republic of Korea | 783.46 (621.96 to 983.77) | 4.08 (3.19 to 5.17) | 1145.25 (1004.73 to 1299.32) | 10.31 (8.91 to 11.87) | 1.77  (1.15 to 2.39) |
| Belgium | 297.79 (251.69 to 348.89) | 10.61 (8.83 to 12.73) | 249.72 (192.98 to 320.64) | 9.13 (7.06 to 11.79) | -0.87  (-1.17 to -0.58) |
| Romania | 660.35 (510.23 to 849.47) | 7.84 (5.95 to 10.19) | 409.51 (323.26 to 512.59) | 9.09 (7.15 to 11.51) | 0.48  (0.41 to 0.54) |
| Latvia | 54.14 (42.59 to 68.12) | 6.88 (5.35 to 8.74) | 35.72 (28.21 to 45.67) | 9.07 (7.1 to 11.74) | 0.54  (0.38 to 0.7) |
| Ireland | 118.76 (93.87 to 151.05) | 7.9 (6.2 to 10.12) | 115.85 (89.65 to 149.11) | 8.23 (6.31 to 10.72) | -0.06  (-0.23 to 0.11) |
| Malta | 8.11 (6.17 to 10.59) | 6.64 (4.95 to 8.76) | 6.43 (5.03 to 8.19) | 7.26 (5.6 to 9.41) | 0.1  (0 to 0.19) |
| Greece | 213.35 (164.86 to 274) | 6.32 (4.87 to 8.2) | 146.97 (116.83 to 183.73) | 6.62 (5.15 to 8.36) | -0.18  (-0.37 to 0.02) |
| Monaco | 0.38 (0.29 to 0.5) | 6.84 (5.17 to 8.98) | 0.51 (0.39 to 0.66) | 6.61 (4.96 to 8.72) | -0.14  (-0.16 to -0.13) |
| Andorra | 1.14 (0.86 to 1.46) | 6.81 (5.1 to 8.87) | 1.19 (0.91 to 1.54) | 6.56 (4.99 to 8.55) | -0.14  (-0.17 to -0.12) |
| Portugal | 157.51 (122.27 to 200.7) | 4.28 (3.31 to 5.52) | 156.23 (125.48 to 195.26) | 6.43 (5.09 to 8.12) | 0.95  (0.58 to 1.31) |
| San Marino | 0.53 (0.4 to 0.68) | 6.61 (5.04 to 8.53) | 0.56 (0.43 to 0.72) | 6.42 (4.85 to 8.35) | -0.14  (-0.15 to -0.13) |
| Turkey | 1114.25 (863.57 to 1422.54) | 3.93 (3.01 to 5.03) | 1687.34 (1314 to 2150.68) | 6.17 (4.75 to 7.94) | 1.37  (1.19 to 1.55) |
| Jordan | 94.19 (73.47 to 119.04) | 4.56 (3.52 to 5.83) | 318.38 (244.92 to 409.85) | 6.1 (4.66 to 7.9) | 1.35  (1.18 to 1.53) |
| Estonia | 25.74 (20.35 to 32.1) | 5.38 (4.2 to 6.74) | 16.77 (12.78 to 21.49) | 6.05 (4.55 to 7.85) | 0.39  (0.36 to 0.42) |
| Israel | 123.7 (97.78 to 156.41) | 5.96 (4.66 to 7.62) | 178.67 (142.72 to 224.59) | 5.57 (4.35 to 7.11) | -0.76  (-1.08 to -0.44) |
| Belarus | 161.37 (123.85 to 206.11) | 5 (3.82 to 6.5) | 106.95 (81.52 to 137.59) | 5.36 (4.07 to 6.99) | 0.29  (0.25 to 0.34) |
| Cyprus | 9.49 (7.22 to 12.34) | 3.53 (2.65 to 4.63) | 15.22 (12.32 to 18.95) | 5.32 (4.26 to 6.72) | 0.82  (0.46 to 1.18) |
| Republic of Moldova | 63.57 (47.94 to 83.32) | 4.13 (3.09 to 5.43) | 43.48 (34.68 to 54.12) | 5.08 (4.03 to 6.38) | 0.7  (0.63 to 0.78) |
| Brunei Darussalam | 4.83 (3.6 to 6.4) | 4.47 (3.29 to 6) | 7.36 (5.52 to 9.84) | 4.83 (3.59 to 6.48) | 0.26  (0.25 to 0.28) |
| Netherlands | 726.81 (624.88 to 847.99) | 15.99 (13.62 to 18.93) | 199.63 (154.09 to 255.54) | 4.58 (3.51 to 5.93) | -3.98  (-4.93 to -3.03) |
| Georgia | 80.01 (60.87 to 104.29) | 4.28 (3.22 to 5.62) | 37.93 (28.67 to 49.19) | 4.3 (3.2 to 5.66) | 0.01  (-0.02 to 0.04) |
| Kazakhstan | 265.02 (197.86 to 347.9) | 4.11 (3.03 to 5.46) | 234.44 (176.82 to 305.82) | 4.27 (3.18 to 5.62) | 0.13  (0.11 to 0.14) |
| Armenia | 49.96 (37.61 to 64.42) | 3.95 (2.95 to 5.14) | 31.7 (24.1 to 41.49) | 4.24 (3.17 to 5.58) | 0.26  (0.24 to 0.28) |
| Azerbaijan | 119.9 (90.54 to 156.84) | 3.9 (2.91 to 5.16) | 122.24 (92.82 to 159.09) | 4.09 (3.08 to 5.35) | 0.24  (0.17 to 0.3) |
| Turkmenistan | 64.26 (48.42 to 83.24) | 3.74 (2.79 to 4.93) | 73 (55.18 to 95.32) | 4.08 (3.08 to 5.38) | 0.33  (0.28 to 0.38) |
| Russian Federation | 1843.49 (1397.4 to 2372.72) | 3.98 (2.99 to 5.2) | 1321.62 (999.32 to 1698.48) | 3.99 (2.99 to 5.21) | 0.01  (0 to 0.02) |
| Uzbekistan | 356.21 (266.84 to 464.29) | 3.74 (2.78 to 4.93) | 500.74 (376.42 to 656.12) | 3.94 (2.94 to 5.24) | 0.15  (0.13 to 0.17) |
| Kyrgyzstan | 74.97 (57.05 to 97.73) | 3.87 (2.9 to 5.1) | 91.71 (69.02 to 120.97) | 3.86 (2.92 to 5.11) | -0.02  (-0.03 to 0) |
| Singapore | 44.31 (33.15 to 58.46) | 3.93 (2.9 to 5.19) | 41.37 (30.96 to 55.72) | 3.86 (2.87 to 5.23) | -0.14  (-0.2 to -0.08) |
| Mongolia | 38.55 (28.86 to 50.77) | 3.66 (2.71 to 4.87) | 39.01 (29.2 to 50.33) | 3.85 (2.84 to 5.07) | 0.17  (0.16 to 0.18) |
| Tajikistan | 88.61 (66.93 to 115.92) | 3.56 (2.66 to 4.74) | 141.03 (106.59 to 187.2) | 3.65 (2.73 to 4.9) | 0.08  (0.06 to 0.11) |
| Kuwait | 32.36 (27.44 to 38.02) | 5.25 (4.41 to 6.22) | 35.3 (26.06 to 46.24) | 3.34 (2.44 to 4.44) | -1.62  (-2.06 to -1.17) |
| Brazil | 2597.49 (2033.62 to 3300.71) | 3.79 (2.94 to 4.87) | 2351.71 (1806.86 to 3043.02) | 3.33 (2.54 to 4.32) | -0.23  (-0.33 to -0.13) |
| Ukraine | 489.72 (367.81 to 643.18) | 3.06 (2.27 to 4.07) | 284.8 (215.1 to 372.37) | 3.1 (2.3 to 4.1) | 0.12  (0.05 to 0.19) |
| Barbados | 3.01 (2.22 to 3.91) | 3.12 (2.29 to 4.09) | 2.51 (1.87 to 3.27) | 2.99 (2.21 to 3.97) | -0.01  (-0.09 to 0.07) |
| United Arab Emirates | 13.78 (10.34 to 18.22) | 2.47 (1.82 to 3.34) | 44.42 (33.32 to 58.92) | 2.86 (2.13 to 3.84) | 0.57  (0.53 to 0.62) |
| Chile | 142.04 (106.95 to 185.29) | 2.57 (1.91 to 3.41) | 151.46 (113.46 to 197.12) | 2.85 (2.11 to 3.76) | 0.47  (-0.08 to 1.02) |
| Bahrain | 4.9 (3.72 to 6.27) | 2.85 (2.15 to 3.7) | 10.27 (7.67 to 13.53) | 2.8 (2.06 to 3.76) | -0.18  (-0.3 to -0.07) |
| Qatar | 3.2 (2.39 to 4.35) | 2.45 (1.8 to 3.33) | 13.34 (10.02 to 17.68) | 2.76 (2.05 to 3.71) | 0.38  (0.31 to 0.44) |
| Saudi Arabia | 187.86 (143.87 to 243.39) | 2.48 (1.87 to 3.28) | 303.85 (228.85 to 397.69) | 2.75 (2.05 to 3.66) | 0.59  (0.43 to 0.75) |
| Egypt | 743.5 (560.7 to 950.91) | 2.89 (2.16 to 3.76) | 1137.36 (838.37 to 1520.07) | 2.74 (1.99 to 3.76) | -0.62  (-0.84 to -0.4) |
| Puerto Rico | 45.81 (37.49 to 56.31) | 3.14 (2.51 to 3.89) | 25.51 (19.98 to 32.01) | 2.64 (2.04 to 3.37) | -0.19  (-0.32 to -0.05) |
| China | 6757.45 (4930.57 to 9142.18) | 1.31 (0.93 to 1.79) | 8284.84 (6242.36 to 10625.62) | 2.59 (1.96 to 3.37) | 2.63  (2.19 to 3.07) |
| Lebanon | 30.15 (22.21 to 41.14) | 2.12 (1.53 to 2.92) | 39.66 (28.99 to 53.4) | 2.53 (1.83 to 3.46) | 0.64  (0.49 to 0.79) |
| Tunisia | 89.02 (65.55 to 119.96) | 2.25 (1.63 to 3.08) | 89.94 (65.9 to 120.6) | 2.53 (1.84 to 3.41) | 0.43  (0.36 to 0.5) |
| Taiwan (Province of China) | 96.57 (71.46 to 126.89) | 1.21 (0.87 to 1.62) | 126.67 (98.07 to 160.44) | 2.5 (1.92 to 3.19) | 3.37  (2.92 to 3.83) |
| Algeria | 317.12 (239.3 to 407.13) | 2.5 (1.86 to 3.25) | 340.65 (252.55 to 460.63) | 2.48 (1.82 to 3.37) | -0.2  (-0.3 to -0.09) |
| Palestine | 21.08 (15.63 to 28.61) | 2.05 (1.5 to 2.78) | 54.7 (39.93 to 73.7) | 2.34 (1.7 to 3.16) | 0.62  (0.54 to 0.69) |
| Oman | 17.27 (12.7 to 22.91) | 2.17 (1.57 to 2.94) | 26.88 (19.81 to 35.77) | 2.33 (1.69 to 3.15) | 0.17  (0.07 to 0.27) |
| Syrian Arab Republic | 130.46 (96.29 to 175.03) | 1.96 (1.43 to 2.67) | 171.09 (125.91 to 231.59) | 2.24 (1.62 to 3.07) | 0.61  (0.47 to 0.75) |
| Sudan | 170.77 (126.55 to 233.86) | 1.75 (1.27 to 2.41) | 410.12 (303.01 to 547.84) | 2.06 (1.49 to 2.8) | 0.61  (0.56 to 0.66) |
| Paraguay | 45.21 (33.16 to 59.04) | 2.45 (1.79 to 3.28) | 57.17 (41.46 to 75.26) | 2.04 (1.45 to 2.73) | -0.86  (-1.01 to -0.71) |
| Bermuda | 0.33 (0.25 to 0.44) | 1.97 (1.44 to 2.64) | 0.27 (0.2 to 0.36) | 2 (1.45 to 2.7) | 0.15  (0.1 to 0.2) |
| Iraq | 144.98 (107.27 to 193.18) | 1.63 (1.18 to 2.21) | 379.57 (273.77 to 515.37) | 1.98 (1.41 to 2.74) | 0.68  (0.59 to 0.77) |
| Libya | 35.57 (26.1 to 48.27) | 1.63 (1.18 to 2.26) | 47.9 (35.01 to 65.11) | 1.93 (1.38 to 2.64) | 0.53  (0.41 to 0.65) |
| United States Virgin Islands | 0.8 (0.59 to 1.07) | 1.85 (1.34 to 2.49) | 0.55 (0.4 to 0.72) | 1.93 (1.39 to 2.59) | 0.23  (0.18 to 0.28) |
| Yemen | 108.67 (79.48 to 147.03) | 1.66 (1.19 to 2.3) | 294.43 (215.1 to 398.07) | 1.91 (1.37 to 2.62) | 0.66  (0.57 to 0.75) |
| Iran (Islamic Republic of) | 511.77 (369.8 to 708.9) | 1.79 (1.28 to 2.51) | 453.61 (329.67 to 627.78) | 1.83 (1.31 to 2.56) | -0.02  (-0.28 to 0.25) |
| Bahamas | 2.26 (1.65 to 3.02) | 1.93 (1.4 to 2.61) | 2.4 (1.73 to 3.26) | 1.82 (1.3 to 2.51) | -0.08  (-0.16 to -0.01) |
| Antigua and Barbuda | 0.45 (0.33 to 0.6) | 1.8 (1.31 to 2.43) | 0.52 (0.38 to 0.71) | 1.82 (1.32 to 2.5) | 0.08  (0.04 to 0.12) |
| Mexico | 755.69 (563.54 to 991.66) | 1.72 (1.26 to 2.29) | 844.06 (635.52 to 1100.82) | 1.79 (1.33 to 2.37) | 0.2  (0.13 to 0.28) |
| Jamaica | 19.56 (14.05 to 26.44) | 1.73 (1.23 to 2.35) | 17.98 (13.14 to 24.21) | 1.75 (1.26 to 2.38) | 0.13  (0.08 to 0.18) |
| Saint Kitts and Nevis | 0.33 (0.24 to 0.44) | 1.72 (1.25 to 2.36) | 0.33 (0.24 to 0.44) | 1.75 (1.26 to 2.39) | 0.15  (0.08 to 0.21) |
| Trinidad and Tobago | 8.67 (6.29 to 11.77) | 1.7 (1.21 to 2.33) | 6.75 (4.88 to 9.17) | 1.72 (1.22 to 2.37) | 0.23  (0.15 to 0.31) |
| Costa Rica | 22.41 (16.45 to 29.97) | 1.68 (1.21 to 2.27) | 26.56 (19.42 to 35.02) | 1.71 (1.25 to 2.29) | 0.04  (0.03 to 0.05) |
| Afghanistan | 98.54 (71.8 to 135.06) | 1.62 (1.15 to 2.23) | 335.27 (245.3 to 460.21) | 1.71 (1.22 to 2.38) | 0.31  (0.25 to 0.38) |
| Cuba | 78.55 (56.53 to 107.68) | 1.75 (1.26 to 2.41) | 48.3 (35.24 to 65.41) | 1.7 (1.22 to 2.32) | -0.02  (-0.07 to 0.03) |
| Saint Lucia | 1.07 (0.77 to 1.44) | 1.58 (1.12 to 2.17) | 0.94 (0.68 to 1.29) | 1.68 (1.2 to 2.34) | 0.27  (0.21 to 0.33) |
| Panama | 19.09 (14.06 to 24.97) | 1.72 (1.26 to 2.28) | 25.8 (18.71 to 34.87) | 1.68 (1.2 to 2.28) | -0.23  (-0.3 to -0.15) |
| Dominica | 0.58 (0.42 to 0.78) | 1.69 (1.22 to 2.29) | 0.4 (0.29 to 0.53) | 1.65 (1.18 to 2.21) | 0.03  (-0.02 to 0.08) |
| Venezuela (Bolivarian Republic of) | 151.5 (110.93 to 200.27) | 1.71 (1.24 to 2.27) | 157.57 (114.44 to 209.75) | 1.59 (1.15 to 2.15) | -0.36  (-0.45 to -0.27) |
| Viet Nam | 328.27 (218.48 to 465.24) | 1.02 (0.66 to 1.45) | 467.72 (325.16 to 652.96) | 1.58 (1.08 to 2.24) | 1.66  (1.31 to 2.02) |
| Grenada | 0.6 (0.43 to 0.82) | 1.51 (1.05 to 2.06) | 0.57 (0.41 to 0.77) | 1.57 (1.11 to 2.16) | 0.15  (0.09 to 0.21) |
| Colombia | 229.69 (166.29 to 308.25) | 1.54 (1.11 to 2.1) | 262.22 (190.05 to 354.31) | 1.57 (1.13 to 2.15) | 0.1  (0.07 to 0.12) |
| Morocco | 169.64 (124.19 to 225.75) | 1.41 (0.99 to 1.89) | 209.25 (154.46 to 278.1) | 1.55 (1.12 to 2.1) | 0.37  (0.34 to 0.41) |
| Saint Vincent and the Grenadines | 0.85 (0.61 to 1.13) | 1.52 (1.08 to 2.05) | 0.6 (0.44 to 0.81) | 1.54 (1.1 to 2.1) | 0.25  (0.19 to 0.31) |
| Suriname | 2.65 (1.9 to 3.59) | 1.52 (1.07 to 2.09) | 3.17 (2.29 to 4.25) | 1.53 (1.09 to 2.08) | 0.17  (0.1 to 0.25) |
| Dominican Republic | 51.94 (37.53 to 71.22) | 1.47 (1.04 to 2.05) | 62.71 (45.01 to 85.67) | 1.51 (1.06 to 2.07) | 0.28  (0.17 to 0.39) |
| Guyana | 5.6 (4.05 to 7.52) | 1.47 (1.04 to 2) | 4.62 (3.37 to 6.27) | 1.5 (1.06 to 2.06) | 0.16  (0.11 to 0.21) |
| Belize | 1.32 (0.96 to 1.79) | 1.4 (0.99 to 1.92) | 2.87 (2.09 to 3.89) | 1.5 (1.08 to 2.06) | 0.34  (0.28 to 0.4) |
| Nicaragua | 26.13 (18.8 to 35.24) | 1.31 (0.92 to 1.78) | 39.79 (28.66 to 52.8) | 1.45 (1.03 to 1.94) | 0.26  (0.21 to 0.3) |
| Uruguay | 16.05 (11.61 to 21.73) | 1.42 (1 to 1.94) | 15.24 (11.17 to 20.5) | 1.44 (1.03 to 1.94) | -0.02  (-0.05 to 0) |
| El Salvador | 36.15 (26.27 to 49.13) | 1.35 (0.96 to 1.84) | 35.41 (25.15 to 48.4) | 1.43 (1.01 to 1.95) | 0.25  (0.21 to 0.28) |
| Sri Lanka | 59.77 (40.98 to 82.81) | 0.79 (0.53 to 1.12) | 105.19 (79.35 to 137.03) | 1.4 (1.03 to 1.85) | 1.91  (1.57 to 2.24) |
| Argentina | 170.15 (122.4 to 231.26) | 1.31 (0.93 to 1.8) | 208.7 (150.8 to 279.15) | 1.36 (0.97 to 1.86) | 0.1  (0.09 to 0.12) |
| Honduras | 30.6 (22.06 to 41.92) | 1.29 (0.91 to 1.77) | 61.44 (43.77 to 83.91) | 1.34 (0.93 to 1.85) | 0.18  (0.15 to 0.2) |
| Ecuador | 50.08 (36.71 to 66.94) | 1.02 (0.73 to 1.38) | 95.42 (71.4 to 123.36) | 1.34 (0.99 to 1.77) | 0.83  (0.72 to 0.94) |
| Guatemala | 46.56 (33.29 to 63.07) | 1.22 (0.86 to 1.68) | 108.31 (77.11 to 145.34) | 1.33 (0.93 to 1.81) | 0.33  (0.3 to 0.36) |
| Democratic People's Republic of Korea | 103.09 (74.01 to 140.04) | 1.31 (0.93 to 1.79) | 99.47 (70.71 to 134.08) | 1.32 (0.92 to 1.79) | 0.01  (-0.04 to 0.06) |
| Haiti | 35.73 (25.71 to 48.05) | 1.22 (0.85 to 1.67) | 67.82 (48.13 to 93.08) | 1.25 (0.87 to 1.75) | 0.14  (0.09 to 0.18) |
| Bhutan | 3.19 (2.2 to 4.59) | 1 (0.67 to 1.46) | 3.61 (2.51 to 5.13) | 1.21 (0.83 to 1.76) | 0.81  (0.72 to 0.91) |
| Bangladesh | 515.48 (362.71 to 729.83) | 0.97 (0.67 to 1.39) | 757.31 (537.8 to 1090.95) | 1.15 (0.8 to 1.67) | 0.43  (0.36 to 0.5) |
| Nepal | 87.34 (60.68 to 124.97) | 0.98 (0.67 to 1.41) | 154.31 (108.07 to 215.66) | 1.1 (0.75 to 1.56) | 0.41  (0.39 to 0.43) |
| Malaysia | 54.52 (38.6 to 74.18) | 0.68 (0.47 to 0.94) | 120 (89.05 to 157.03) | 1.05 (0.77 to 1.39) | 1.29  (1.1 to 1.49) |
| India | 4113.85 (2910 to 5857.59) | 1.08 (0.76 to 1.54) | 5682.38 (4025.66 to 8029.36) | 0.99 (0.68 to 1.41) | -0.11  (-0.3 to 0.08) |
| Peru | 102.67 (71.29 to 142.55) | 0.98 (0.67 to 1.38) | 121.19 (85.75 to 168.37) | 0.97 (0.67 to 1.36) | -0.09  (-0.13 to -0.05) |
| Pakistan | 453.23 (319.02 to 642.38) | 0.83 (0.56 to 1.19) | 1027.16 (719.06 to 1465.05) | 0.93 (0.63 to 1.34) | 0.45  (0.42 to 0.47) |
| Gabon | 3.69 (2.51 to 5.17) | 0.81 (0.54 to 1.14) | 7.12 (4.86 to 10.04) | 0.92 (0.61 to 1.3) | 0.42  (0.39 to 0.44) |
| Cabo Verde | 1.34 (0.9 to 1.87) | 0.78 (0.52 to 1.11) | 1.97 (1.37 to 2.76) | 0.9 (0.61 to 1.28) | 0.49  (0.45 to 0.52) |
| Botswana | 5.18 (3.48 to 7.37) | 0.75 (0.49 to 1.08) | 8.34 (5.71 to 11.72) | 0.89 (0.59 to 1.25) | 0.44  (0.34 to 0.53) |
| Namibia | 5.51 (3.71 to 8) | 0.76 (0.5 to 1.11) | 9.36 (6.36 to 13.11) | 0.88 (0.57 to 1.24) | 0.45  (0.38 to 0.51) |
| Bolivia (Plurinational State of) | 26.19 (18.2 to 36.71) | 0.86 (0.59 to 1.22) | 41.09 (29.06 to 55.96) | 0.87 (0.6 to 1.21) | -0.02  (-0.05 to 0.01) |
| Ghana | 56.11 (37.44 to 78.44) | 0.78 (0.51 to 1.11) | 126.26 (86.1 to 178.3) | 0.87 (0.59 to 1.23) | 0.38  (0.35 to 0.42) |
| Equatorial Guinea | 1.22 (0.8 to 1.72) | 0.61 (0.39 to 0.89) | 6.74 (4.73 to 9.32) | 0.85 (0.58 to 1.2) | 1.31  (1.19 to 1.43) |
| Sao Tome and Principe | 0.48 (0.33 to 0.69) | 0.77 (0.51 to 1.11) | 0.87 (0.61 to 1.21) | 0.85 (0.58 to 1.22) | 0.37  (0.32 to 0.42) |
| Congo | 9.77 (6.73 to 13.79) | 0.79 (0.53 to 1.12) | 19.81 (13.54 to 27.88) | 0.84 (0.56 to 1.2) | 0.26  (0.2 to 0.33) |
| Mauritania | 6.82 (4.63 to 9.67) | 0.7 (0.46 to 1.01) | 16.91 (11.67 to 23.32) | 0.83 (0.55 to 1.17) | 0.44  (0.36 to 0.51) |
| Mauritius | 3.04 (2.01 to 4.28) | 0.67 (0.42 to 0.96) | 3.06 (2.12 to 4.32) | 0.81 (0.54 to 1.15) | 0.61  (0.58 to 0.64) |
| South Africa | 134.27 (91.67 to 188.98) | 0.76 (0.51 to 1.08) | 162.4 (110.95 to 229.73) | 0.81 (0.55 to 1.15) | 0.31  (0.25 to 0.36) |
| Seychelles | 0.23 (0.15 to 0.32) | 0.7 (0.45 to 1.01) | 0.24 (0.17 to 0.34) | 0.81 (0.54 to 1.14) | 0.41  (0.36 to 0.45) |
| Eswatini | 3.03 (2.06 to 4.32) | 0.71 (0.47 to 1.02) | 4.21 (2.87 to 5.81) | 0.78 (0.52 to 1.1) | 0.35  (0.29 to 0.4) |
| Cameroon | 34.59 (23.37 to 49.18) | 0.7 (0.47 to 1) | 113.38 (76.47 to 159.04) | 0.77 (0.51 to 1.1) | 0.34  (0.3 to 0.37) |
| Zimbabwe | 40.8 (27.64 to 58.16) | 0.73 (0.48 to 1.05) | 54.57 (36.77 to 78.03) | 0.74 (0.49 to 1.05) | 0.04  (-0.01 to 0.1) |
| Lesotho | 6.07 (4.07 to 8.67) | 0.67 (0.44 to 0.96) | 7 (4.8 to 9.93) | 0.74 (0.49 to 1.06) | 0.34  (0.29 to 0.39) |
| Djibouti | 1.57 (1.05 to 2.26) | 0.63 (0.41 to 0.92) | 3.48 (2.35 to 4.9) | 0.73 (0.48 to 1.04) | 0.67  (0.58 to 0.76) |
| Zambia | 26.46 (17.79 to 37.41) | 0.62 (0.41 to 0.89) | 68 (46.01 to 94.23) | 0.73 (0.49 to 1.03) | 0.56  (0.44 to 0.69) |
| Angola | 30.16 (20.22 to 43.12) | 0.62 (0.4 to 0.89) | 105.45 (72.29 to 147.08) | 0.72 (0.47 to 1.02) | 0.43  (0.38 to 0.49) |
| Cook Islands | 0.06 (0.04 to 0.08) | 0.67 (0.44 to 0.97) | 0.04 (0.03 to 0.06) | 0.72 (0.48 to 1.03) | 0.16  (0.13 to 0.19) |
| Guam | 0.36 (0.24 to 0.52) | 0.68 (0.45 to 0.99) | 0.42 (0.29 to 0.59) | 0.72 (0.48 to 1.02) | 0.15  (0.09 to 0.2) |
| Nigeria | 262.21 (175.45 to 369.78) | 0.61 (0.4 to 0.88) | 781.32 (535.13 to 1106.05) | 0.71 (0.47 to 1.01) | 0.54  (0.48 to 0.59) |
| Togo | 13.28 (9.04 to 18.46) | 0.72 (0.47 to 1.03) | 26.32 (17.66 to 37.66) | 0.71 (0.46 to 1.03) | -0.03  (-0.07 to 0) |
| Northern Mariana Islands | 0.12 (0.08 to 0.17) | 0.72 (0.47 to 1.03) | 0.11 (0.08 to 0.16) | 0.71 (0.47 to 1.02) | -0.15  (-0.21 to -0.09) |
| Gambia | 3.2 (2.08 to 4.46) | 0.68 (0.43 to 0.97) | 8.15 (5.53 to 11.7) | 0.7 (0.46 to 1.02) | 0.04  (-0.03 to 0.1) |
| United Republic of Tanzania | 85.12 (57.32 to 120.63) | 0.65 (0.43 to 0.93) | 192.35 (128.1 to 272.47) | 0.69 (0.45 to 0.99) | 0.19  (0.14 to 0.23) |
| Senegal | 24.4 (16.45 to 34.37) | 0.65 (0.43 to 0.95) | 51.91 (34.89 to 73.54) | 0.69 (0.45 to 0.99) | 0.14  (0.09 to 0.19) |
| American Samoa | 0.14 (0.09 to 0.2) | 0.65 (0.42 to 0.94) | 0.18 (0.12 to 0.25) | 0.69 (0.45 to 0.99) | 0.14  (0.1 to 0.18) |
| Benin | 13.83 (9.47 to 19.78) | 0.62 (0.41 to 0.9) | 42.61 (28.7 to 60.01) | 0.68 (0.45 to 0.98) | 0.26  (0.23 to 0.29) |
| Rwanda | 21.3 (14.06 to 30.26) | 0.61 (0.39 to 0.87) | 42.57 (28.4 to 59.96) | 0.68 (0.44 to 0.98) | 0.44  (0.36 to 0.52) |
| Maldives | 0.6 (0.39 to 0.87) | 0.55 (0.34 to 0.8) | 0.89 (0.6 to 1.25) | 0.67 (0.44 to 0.95) | 0.71  (0.67 to 0.75) |
| Comoros | 1.49 (1 to 2.09) | 0.63 (0.41 to 0.9) | 2.11 (1.42 to 2.96) | 0.67 (0.44 to 0.95) | 0.27  (0.23 to 0.32) |
| Sierra Leone | 9.92 (6.5 to 14.15) | 0.61 (0.4 to 0.88) | 27.32 (18.43 to 38.68) | 0.66 (0.43 to 0.96) | 0.25  (0.23 to 0.28) |
| Guinea-Bissau | 3.05 (2.04 to 4.34) | 0.61 (0.4 to 0.88) | 6.25 (4.21 to 8.98) | 0.66 (0.44 to 0.97) | 0.28  (0.24 to 0.32) |
| Thailand | 121.71 (77.22 to 178.93) | 0.48 (0.29 to 0.73) | 116.27 (78.11 to 161.91) | 0.65 (0.43 to 0.94) | 1.06  (0.97 to 1.15) |
| Democratic Republic of the Congo | 122.98 (80.78 to 173.51) | 0.67 (0.43 to 0.96) | 285.92 (191.3 to 416.29) | 0.65 (0.42 to 0.96) | -0.2  (-0.35 to -0.06) |
| Kenya | 74.93 (49.99 to 106.06) | 0.61 (0.4 to 0.88) | 168.55 (114.98 to 238.68) | 0.65 (0.43 to 0.93) | 0.03  (-0.02 to 0.08) |
| Guinea | 16.41 (10.62 to 23.55) | 0.61 (0.39 to 0.89) | 40.26 (27.02 to 56.06) | 0.65 (0.42 to 0.93) | 0.28  (0.22 to 0.34) |
| Niue | 0.01 (0 to 0.01) | 0.62 (0.39 to 0.89) | 0 (0 to 0.01) | 0.65 (0.42 to 0.91) | 0.15  (0.1 to 0.2) |
| Tokelau | 0 (0 to 0.01) | 0.55 (0.35 to 0.8) | 0 (0 to 0) | 0.65 (0.42 to 0.92) | 0.63  (0.56 to 0.69) |
| Palau | 0.04 (0.03 to 0.06) | 0.63 (0.4 to 0.89) | 0.03 (0.02 to 0.05) | 0.65 (0.42 to 0.92) | -0.01  (-0.07 to 0.04) |
| Eritrea | 8.89 (5.92 to 12.61) | 0.59 (0.38 to 0.84) | 21.46 (14.41 to 30.41) | 0.64 (0.42 to 0.91) | 0.26  (0.21 to 0.3) |
| Liberia | 6.09 (4.11 to 8.63) | 0.64 (0.42 to 0.93) | 15.37 (10.23 to 21.88) | 0.64 (0.42 to 0.93) | 0.25  (0.13 to 0.36) |
| Madagascar | 35.45 (24.01 to 50.04) | 0.6 (0.4 to 0.86) | 83.16 (56.21 to 118.48) | 0.63 (0.41 to 0.9) | 0.2  (0.17 to 0.23) |
| South Sudan | 18.26 (11.98 to 25.81) | 0.6 (0.39 to 0.86) | 32.11 (21.53 to 45.53) | 0.63 (0.41 to 0.9) | 0.13  (0.11 to 0.15) |
| Uganda | 48.77 (31.99 to 69.85) | 0.56 (0.36 to 0.81) | 139.24 (93.45 to 196.95) | 0.63 (0.41 to 0.91) | 0.65  (0.56 to 0.75) |
| Mali | 22.47 (14.58 to 32.19) | 0.57 (0.36 to 0.84) | 69.43 (46.98 to 98.78) | 0.62 (0.4 to 0.89) | 0.26  (0.22 to 0.31) |
| Chad | 16.27 (10.57 to 23.5) | 0.57 (0.37 to 0.84) | 52.18 (34.78 to 75.24) | 0.62 (0.4 to 0.91) | 0.28  (0.26 to 0.31) |
| Burkina Faso | 26.36 (17.12 to 38.24) | 0.57 (0.36 to 0.84) | 69.3 (45.65 to 98.95) | 0.62 (0.4 to 0.91) | 0.35  (0.29 to 0.41) |
| Tonga | 0.3 (0.19 to 0.43) | 0.58 (0.37 to 0.84) | 0.28 (0.19 to 0.4) | 0.6 (0.39 to 0.86) | -0.02  (-0.08 to 0.04) |
| Malawi | 26.19 (16.94 to 38.16) | 0.57 (0.36 to 0.83) | 61.53 (40.75 to 87.78) | 0.6 (0.38 to 0.86) | 0.26  (0.23 to 0.29) |
| Nauru | 0.03 (0.02 to 0.04) | 0.58 (0.38 to 0.84) | 0.03 (0.02 to 0.04) | 0.6 (0.38 to 0.86) | -0.1  (-0.29 to 0.09) |
| Philippines | 159.84 (104.98 to 226.38) | 0.52 (0.33 to 0.75) | 275.2 (184.46 to 383.31) | 0.59 (0.39 to 0.84) | 0.41  (0.33 to 0.48) |
| Central African Republic | 7.75 (5.19 to 11.1) | 0.61 (0.39 to 0.88) | 15.67 (10.33 to 22.27) | 0.59 (0.38 to 0.86) | -0.03  (-0.04 to -0.02) |
| Samoa | 0.53 (0.34 to 0.76) | 0.59 (0.37 to 0.84) | 0.61 (0.39 to 0.87) | 0.58 (0.37 to 0.83) | -0.25  (-0.33 to -0.16) |
| Myanmar | 90.73 (57.94 to 131.33) | 0.48 (0.29 to 0.7) | 123.8 (80.98 to 176.39) | 0.58 (0.37 to 0.84) | 0.72  (0.67 to 0.76) |
| Mozambique | 34.3 (22.64 to 49.17) | 0.53 (0.34 to 0.77) | 87.33 (56.48 to 127.24) | 0.58 (0.37 to 0.84) | 0.31  (0.28 to 0.33) |
| Niger | 21.69 (14.13 to 31.34) | 0.57 (0.36 to 0.83) | 70.38 (45.87 to 101.62) | 0.58 (0.38 to 0.85) | 0.05  (0.01 to 0.08) |
| Timor-Leste | 1.57 (0.99 to 2.29) | 0.46 (0.28 to 0.68) | 3.96 (2.59 to 5.69) | 0.57 (0.37 to 0.83) | 0.88  (0.81 to 0.95) |
| Fiji | 1.88 (1.23 to 2.73) | 0.54 (0.34 to 0.77) | 1.97 (1.3 to 2.83) | 0.57 (0.37 to 0.84) | 0.16  (0.07 to 0.24) |
| Cambodia | 22.67 (14.42 to 32.2) | 0.47 (0.29 to 0.68) | 37.06 (24.5 to 53.46) | 0.57 (0.36 to 0.83) | 0.63  (0.59 to 0.66) |
| Lao People's Democratic Republic | 9.18 (5.88 to 13.53) | 0.46 (0.28 to 0.69) | 16.93 (11.29 to 24.05) | 0.56 (0.35 to 0.81) | 0.65  (0.61 to 0.68) |
| Ethiopia | 127.98 (85.19 to 183.72) | 0.51 (0.33 to 0.74) | 314.91 (207.04 to 453.69) | 0.56 (0.36 to 0.82) | 0.35  (0.3 to 0.4) |
| Indonesia | 402.73 (259.72 to 581.09) | 0.46 (0.28 to 0.67) | 531.22 (351.01 to 751.56) | 0.54 (0.35 to 0.78) | 0.59  (0.49 to 0.68) |
| Micronesia (Federated States of) | 0.29 (0.18 to 0.41) | 0.53 (0.33 to 0.77) | 0.26 (0.17 to 0.37) | 0.54 (0.34 to 0.79) | 0.01  (-0.06 to 0.07) |
| Marshall Islands | 0.12 (0.08 to 0.17) | 0.51 (0.31 to 0.74) | 0.13 (0.09 to 0.19) | 0.54 (0.34 to 0.78) | 0.1  (0.05 to 0.15) |
| Tuvalu | 0.02 (0.01 to 0.03) | 0.52 (0.33 to 0.77) | 0.03 (0.02 to 0.04) | 0.54 (0.35 to 0.78) | -0.01  (-0.05 to 0.04) |
| Burundi | 14.09 (9.21 to 20.43) | 0.55 (0.35 to 0.81) | 30.8 (20.43 to 44.14) | 0.52 (0.33 to 0.77) | 0.11  (0.02 to 0.21) |
| Vanuatu | 0.35 (0.22 to 0.51) | 0.49 (0.3 to 0.72) | 0.67 (0.43 to 0.97) | 0.5 (0.31 to 0.74) | 0  (-0.06 to 0.07) |
| Kiribati | 0.16 (0.1 to 0.23) | 0.48 (0.29 to 0.72) | 0.24 (0.16 to 0.34) | 0.48 (0.3 to 0.68) | -0.19  (-0.26 to -0.12) |
| Somalia | 18.63 (12.13 to 26.64) | 0.49 (0.32 to 0.72) | 49.98 (32.38 to 71.98) | 0.47 (0.30 to 0.70) | -0.1  (-0.13 to -0.08) |
| Papua New Guinea | 8.58 (5.31 to 12.44) | 0.44 (0.26 to 0.65) | 19.14 (12.37 to 27.57) | 0.45 (0.28 to 0.65) | 0.02  (-0.02 to 0.06) |
| Solomon Islands | 0.79 (0.49 to 1.15) | 0.44 (0.27 to 0.65) | 1.39 (0.88 to 1.99) | 0.45 (0.28 to 0.66) | -0.03  (-0.09 to 0.03) |
